# Functional traits of plants and pollinators explain resource overlap between honeybees and wild pollinators

**DOI:** 10.1007/s00442-022-05151-6

**Published:** 2022-04-05

**Authors:** Andree Cappellari, Giovanna Bonaldi, Maurizio Mei, Dino Paniccia, Pierfilippo Cerretti, Lorenzo Marini

**Affiliations:** 1 Department of Agronomy, Food, Natural resources, Animals and Environment (DAFNAE), University of Padua, Legnaro, Padua Italy; 2 Department of Biology and Biotechnology “Charles Darwin”, Sapienza University of Rome, Rome, Italy; 3 Via Colle 13, 03100 Frosinone, Italy

**Keywords:** *Apis mellifera*, Competition, Foraging behaviour, Plant–pollinator networks, Trait similarity

## Abstract

Managed and wild pollinators often cohabit in both managed and natural ecosystems. The western honeybee, *Apis mellifera*, is the most widespread managed pollinator species. Due to its density and behaviour, it can potentially influence the foraging activity of wild pollinators, but the strength and direction of this effect are often context-dependent. Here, we observed plant–pollinator interactions in 51 grasslands, and we measured functional traits of both plants and pollinators. Using a multi-model inference approach, we explored the effects of honeybee abundance, temperature, plant functional diversity, and trait similarity between wild pollinators and the honeybee on the resource overlap between wild pollinators and the honeybee. Resource overlap decreased with increasing honeybee abundance only in plant communities with high functional diversity, suggesting a potential diet shift of wild pollinators in areas with a high variability of flower morphologies. Moreover, resource overlap increased with increasing trait similarity between wild pollinators and the honeybee. In particular, central-place foragers of family Apidae with proboscis length similar to the honeybee exhibited the highest resource overlap. Our results underline the importance of promoting functional diversity of plant communities to support wild pollinators in areas with a high density of honeybee hives. Moreover, greater attention should be paid to areas where pollinators possess functional traits similar to the honeybee, as they are expected to be more prone to potential competition with this species.

**Supplementary Information:**

The online version contains supplementary material available at 10.1007/s00442-022-05151-6.

## Introduction

As a managed and super-generalist pollinator, the western honeybee, *Apis mellifera* Linnaeus, plays a fundamental role in the pollination of both crops (Garibaldi et al. 2013) and wild plants (Hung et al. 2018). However, managed honeybees might adversely impact wild pollinator communities, as they are often extremely abundant, have a prolonged flight season, and tend to forage on the most abundant and rewarding floral resources (Goulson 2003). Nevertheless, observed effects are often idiosyncratic and seem to depend on local conditions, on the composition of wild pollinator communities, and on the different methodological approaches adopted (Goulson 2003; Cane and Tepedino 2017; Mallinger et al. 2017).

**Fig. 1:**
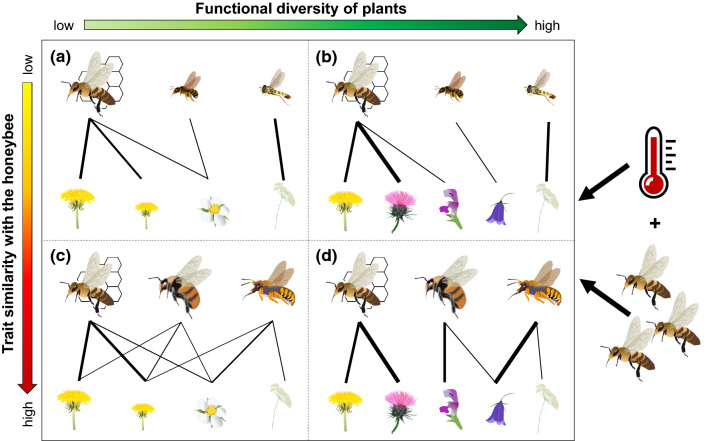
Expected effects of functional diversity of plant community and trait similarity between wild pollinator community and the honeybee on plant–pollinator interactions. We hypothesise that: **a** in sites with a low functional diversity of plant community and a low trait similarity between wild pollinator community and the honeybee, the resource overlap between wild pollinators and the honeybee would be generally low, as pollinator species with functional traits different from those of the honeybee would exploit different resources; **b** in sites with a high functional diversity of plant community and a low trait similarity between wild pollinator community and the honeybee, the resource overlap would be even lower, as pollinator species would spread on different floral resources; **c** in sites with a low functional diversity of plant community and a high trait similarity between wild pollinator community and the honeybee, pollinator species would share an important portion of plants with the honeybee, therefore, resulting in a high resource overlap; **d** in sites with a high functional diversity of plant community and a high trait similarity between wild pollinator community and the honeybee, the resource overlap would decrease, as pollinator species would have much more resources to forage on. Increasing honeybee abundance and higher temperatures would intensify the observed effects

Against this background, functional traits of both plants and pollinators can help to identify the likelihood, strength and direction of the interactions between managed and wild pollinators (Violle et al. 2007; Eklöf et al. 2013; Schleuning et al. 2015; Bergamo et al. 2020). Floral morphological traits are fundamental in shaping plant–pollinator interactions (Junker et al. 2013). Plant species with greater flower size and longer flowering periods are usually more generalist, being attractive to many pollinator species, while flowers with deep corolla are usually accessible only to a few specialized pollinator species (Lázaro et al. 2020). Although the effect of functional diversity of plant communities on pollinators is still debated (Fornoff et al. 2017; Uyttenbroeck et al. 2017; Goulnik et al. 2020), one expectation is that increased functional diversity should reduce the plant resource overlap between wild pollinators and a dominant species such as the honeybee by providing a larger number of alternative nectar and pollen resources (Fig. 1).

Similarly, pollinator traits can affect both how pollinators interact with plant species and how they interact with each other (Albrecht et al. 2012; Garibaldi et al. 2015; Woodcock et al. 2019). In particular, the competition of wild pollinators with honeybees in areas with a high abundance of managed pollinators could be stronger for central-place foragers, which are forced to collect pollen and nectar near to their nest (Walther-Hellwig et al. 2006), and for oligolectic pollinators, which have a limited ability to shift to alternative resources (Cane and Tepedino 2017). On the contrary, large-sized pollinators with longer proboscis usually have a larger diet breadth, as they are able to exploit a wider range of resources compared to smaller ones (Greenleaf et al. 2007; Lara-Romero et al. 2019). Hence, we expect that a high trait similarity between wild pollinators and the honeybee should increase their resource overlap (Fig. 1).

Environmental variables can also have a strong effect on species phenology and behaviour. Air temperature and weather, in particular, modulate the activity of pollinators (Trøjelsgaard and Olesen 2013; Giannini et al. 2015). For example, bumblebees are often active at low temperatures and under unfavourable weather conditions (Goulson 2010), while butterflies are strongly negatively affected by low air temperatures (Abrahamczyk et al. 2011). Honeybees are more sensitive to low temperatures than many wild pollinators (Jaffé et al. 2010), so potential competition between wild pollinators and honeybees should be more severe at high temperatures (Fig. 1).

A promising approach to elucidate potential mechanisms shaping the interactions between plants and pollinators is the use of network tools integrated with functional trait analysis. Here, we investigated how functional traits of both plants and pollinators, together with the abundance of honeybees and temperature, affected the foraging behaviour of wild pollinators. In particular, this study aimed to explore how functional richness and dispersion of plant communities influenced the resource overlap between wild pollinators and the honeybee, also testing the effect of trait similarity between wild pollinators and the honeybee. We observed plant–pollinator networks in 51 grasslands in Northern Italy and computed the resource overlap between each wild pollinator species and the honeybee. We calculated functional richness and functional dispersion of plant communities using flower corolla length, flower colour, and flower shape, while trait similarity between wild pollinators and the honeybee was calculated using proboscis length, body size, type of foraging range, and taxonomic family.

## Materials and methods

### Sampling design

Fieldwork was carried out in 51 grasslands in Northern Italy (Alps and Prealps), approximately 50 × 30 m in size. Grasslands were selected across a steep elevational gradient ranging from 150 to 2100 m a.s.l., and had a wide range of honeybee abundance (Table S1, Fig. S1). The selection of the sites was adjusted during the sampling season to have statistical independence between temperature and honeybee abundance (Pearson’s correlation = 0.11, *p *value = 0.41). Each sampling site was at least 0.53 km from the nearest one (mean = 4.60 km). We were not able to determine the exact number of beehives near the sampling sites, but the mean density in the study area was c. 5 beehives per km^2^ (data provided by the National Data Bank of the Zootechnical Registry established by the Ministry of Health at the National Service Centre of the “G. Caporale” Institute of Teramo).

### Sampling of ecological interactions

Between May and September 2019, we observed plant–pollinator interactions in the selected sites. Sampling occurred between 9:00 and 17:00 only with air temperature > 15 °C, low wind, no rain, and cloud coverage < 70%. Each site was visited only once. At each site, we identified all flowering plant species and assessed their relative abundance. All the individuals of each plant species were then observed for 15 min in total, during which all hymenopterans and dipterans touching the reproductive parts of flowers were counted and collected. Both plant and pollinator species were identified in the field when possible, otherwise, plants were collected and prepared in a herbarium, while pollinators were placed in vials filled with 70% ethanol. Later identification was entrusted to experts (Filippo Prosser and LM for plants, and AC, MM, DP, and PC for pollinators). During the sampling, we also measured the air temperature using a Tinytag Plus 2 TGP-4017 data logger.

### Resource overlap between wild pollinators and the honeybee

Starting from the observed interactions, we built 51 bipartite plant–pollinator networks, one for each sampling site. For each network, we calculated the resource overlap between each wild pollinator species (i.e., excluding the honeybee) and the honeybee using Morisita’s index (Morisita 1959) in the R package *spaa* (Zhang 2016). The index ranges from 0 to 1, with increasing values indicating an increase in the plant resource overlap between the two pollinator species. In each network, we then calculated the community weighted mean (hereafter, CWM) resource overlap between wild pollinators and the honeybee as the mean resource overlap value of all wild pollinator species weighted by their abundance. We used CWM resource overlap instead of resource overlap values of single species as no model using species as replicates met statistical assumptions, even after changing the distribution or transforming the variables. All analyses were performed using R version 3.6.1 (R Core Team 2019).

### Functional traits of plant species

For each flowering plant species, we measured flower corolla length with a calliper, and recorded flower type after Kugler (1970) and flower colour (Table S2). These are among the most important morphological traits for the definition of pollinator feeding niches: flower colour affects the attractiveness and selectivity of flowers, while flower type and corolla length determine the accessibility of flowers to pollinators (Junker et al. 2013). We then calculated two indices of functional diversity of plant communities for each network, i.e., the standardized functional richness and the functional dispersion, which provide complementary information (Villéger et al. 2008; Laliberté and Legendre 2010). First, for each network, we built a Euclidean distance matrix by projecting flowering plant species into a three-dimensional trait space with each axis corresponding to a functional trait. The distance matrix was analysed through Principal Coordinate Analysis (PCoA), and the PCoA axes were then used as new combined traits to compute the functional diversity indices. Categorical variables were transformed into dummy variables (i.e., binary). Functional richness measures the functional space filled by the plant community, i.e., the volume of the convex hull. For each network, we standardized the index value by the “global” functional richness, including all plant species in all networks (Laliberté et al. 2014). Its value ranges from 0 to 1, with increasing values of the index indicating an increase in community functional richness. Functional dispersion additionally takes into account the relative abundance of plant species. The index represents the dispersion of plant species in the trait space, i.e., the distance of species to the centroid of all species in the community, weighted by their relative abundance. Its value ranges from 0 to infinity, with increasing values indicating an increase in functional dispersion, i.e., a strong difference in traits between dominant plant species and low abundant ones. Both indices were calculated using the R package FD (Laliberté et al. 2014).

### Functional traits of pollinator species

For each pollinator species, we selected one to four individuals, depending on the availability, and extracted the proboscis which was measured along with total body length (body size). We derived from the literature two additional traits: type of foraging range (two classes: central-place forager, for species which build a nest, and non-central-place forager), and taxonomic family (Table S3; Additional References in ESM). As for corolla shape and length, proboscis length and body size affect the way a pollinator species can exploit a floral resource. The type of foraging range does not directly influence resource selection, but it determines how far pollinators can travel to collect pollen and nectar. Finally, the taxonomic family is often linked to floral preferences or particular mouthpart morphology. Using these traits, we estimated the trait similarity between each wild pollinator species and the honeybee using Gower’s similarity coefficient (Gower 1971) as described by Podani (1999), calculated using the R package FD (Laliberté et al. 2014). For each site, we then determined the CWM trait similarity between the community of wild pollinators and the honeybee by calculating the mean trait similarity value of all wild pollinator species (i.e., excluding the honeybee) weighted by their abundance.

### Potential collinearity between predictors

Before performing the statistical analyses described below, we analysed potential collinearity in our data by computing the variance inflation factors (VIFs) using the R package car (John and Weisberg 2019). Plant species richness and standardized functional richness of plant community were strongly correlated (Pearson’s correlation = 0.876, *p *value < 0.001), as well as temperature and elevation (Pearson’s correlation = 0.751, *p *value < 0.001). We, therefore, chose to build our models using plant standardized functional richness and temperature as explanatory variables. Functional traits of pollinators were also correlated with each other (Table S4, Fig. S2), so their effect on resource overlap was analysed separately. The explanatory variables of the six global models described in the next paragraph fitted without the interactions had VIFs < 1.5, indicating low collinearity.

### Statistical analyses

For the statistical analyses, we followed an information-theoretic approach (Burnham and Anderson 2002), which allows comparing the fit of a set of models rather than selecting one single best model based on *p* values. The first global model (Model 1) included resource overlap between wild pollinator community and the honeybee as response variable, and the main effects of honeybee abundance, temperature, standardized functional richness of plant community, and trait similarity between wild pollinator community and the honeybee as explanatory variables. The model also included all the interactions that could strongly affect the resource overlap, i.e., the two-way interactions between honeybee abundance and plant standardized functional richness, between honeybee abundance and trait similarity between wild pollinator community and the honeybee, between plant standardized functional richness and trait similarity between wild pollinator community and the honeybee, and the three-way interaction between honeybee abundance, plant standardized functional richness and trait similarity between wild pollinator community and the honeybee. The structure of the second model (Model 2) was similar, but standardized functional richness of plant community was replaced by functional dispersion of plant community.

Second, we explored the effect of single pollinator traits on resource overlap. We, therefore, built four linear mixed-effect models, one for proboscis length (Model 3), one for body size (Model 4), one for type of foraging range (Model 5), and one for taxonomic family (Model 6). Proboscis length and body size of wild pollinators were categorized according to trait values of the honeybee, which possesses a proboscis of c. 5 mm and body size of c. 12 mm. Proboscis length categories for wild pollinators were: proboscis shorter than the honeybee < 3.9 mm, proboscis similar to the honeybee = 4–6.9 mm, and proboscis longer than the honeybee > 7 mm. Body size categories for wild pollinators were: smaller than the honeybee < 7.9 mm, similar to the honeybee = 8–14.9 mm, and larger than the honeybee > 15 mm. We categorized continuous trait variables due to the poor outcome of model residual diagnostics using traits as continuous variables. For taxonomic family, we aggregated families with less than ten collected individuals, i.e., Cimbicidae, Megalodontesidae, Melittidae, and Scoliidae. For each network, we calculated the CWM resource overlap between wild pollinators and the honeybee for each trait category, e.g., for body size, we had one value of CWM resource overlap for wild pollinators smaller than the honeybee, one for wild pollinators similar in size, and one for wild pollinators larger than the honeybee. Each global model included honeybee abundance, temperature, trait category, and the interaction between honeybee abundance and trait category as explanatory variables, and network identity as random factor. In all models described above, the continuous explanatory variables were scaled to mean 0 and standard deviation 1 to make slopes comparable (Gelman 2008). For a summary of the six global models, see Table S5.

Within each set, models were ordered based on their second-order Akaike information criterion corrected for small sample size (AICc), with lower values indicating models that better fit the data. For each model, we calculated the ΔAICc, i.e., the difference between the model AICc and the lowest AICc of the entire set of models (with the best model having ΔAICc = 0), and the Akaike model weight, which indicates the probability that the model is the best one. As a measure of goodness-of-fit, we estimated the *R*^2^. Lastly, we calculated the model-averaged partial coefficient for each explanatory variable using all models within each set and estimated the 95% confidence intervals around model-averaged partial coefficients. We presented in the tables all models with ΔAICc < 6 (Harrison et al. 2018). All multi-model analyses were conducted using the R package MuMIn (Barton 2020).

Lastly, we tested for potential spatial autocorrelation of residuals of all models using Moran’s I in the R package *ape* (Paradis and Schliep 2019). The analyses highlighted no spatial autocorrelation in any of the model (Model 1 *p* value = 0.692; Model 2 *p* value = 0.478; Model 3 *p* value = 0.336; Model 4 *p* value = 0.842; Model 5 *p* value = 0.539; Model 6 *p* value = 0.075).

### Methodological considerations

In this study, we opted to sample many sites with a single visit, as we wanted to include a wide range of plant and pollinator functional traits and temperatures. In network ecology, it is common practice to aggregate data collected in multiple sampling events within a single plant–pollinator network (e.g., Montero-Castaño and Vilà 2017; Norfolk et al. 2018; Valido et al. 2019). However, this operation can potentially create artificial species assemblages, i.e., cumulative communities composed of species observed in different days, weeks or seasons, often with non-overlapping phenology (CaraDonna et al. 2020; Schwarz et al. 2020). Using single visit networks, we aimed at exploring the realized interactions between co-occurring individuals of honeybees and wild pollinators, rather than achieving high sampling completeness of pollinator species or interactions. Our interactions can, therefore, be interpreted as short-term, behavioural responses.

## Results

### General results

Across the 51 networks combined, we observed 262 plant species (Table S2) and 325 pollinator species or morphospecies (Table S3), for a total of 10,841 pollinator visits to flowers. During the 255 h of observation, we recorded 1497 unique plant–pollinator interactions. We identified to the species level 99% of collected wild pollinators (Table S3). We observed an average of 81 wild pollinator individuals (min = 16, max = 332), and of 24 pollinator species (min = 9, max = 49) per site (Table S1). The honeybee was found in all sites and was the most abundant pollinator with 6718 collected individuals (min = 2, max = 768, mean = 132), and the most generalist one, visiting 111 flowering plant species. Other common, abundant and generalist species were *Eristalis tenax* (Linnaeus), a hoverfly species found at 39 sites with 597 individuals that visited 76 flowering plant species, *Bombus pascuorum* (Scopoli), a bumblebee species found at 35 sites with 411 individuals that visited 45 flowering plant species, and *Sphaerophoria scripta* (Linnaeus), a hoverfly species found at 37 sites with 366 individuals that visited 77 flowering plant species. Pollinator proboscis length ranged from 0.4 mm for *Entomognathus brevis* (Vander Linden) to 16 mm for *Bombus gerstaeckeri* Morawitz, while body length ranged from 4 mm for *Hylaeus taeniolatus* Förster and *H. imparilis* Förster to 22.5 mm for *Xylocopa violacea* Linnaeus (Table S3).

We observed an average of 20 flowering plant species (min = 8, max = 35) per site (Table S1). The most frequently visited species were *Rubus* sp. L. (931 total visits, 97% by the honeybee), *Centaurea nigrescens* Willd. (823 total visits, 84% by the honeybee), and *Epilobium angustifolium* L. (560 total visits, 93% by the honeybee), while the species most frequently visited only by wild pollinators were *Galeopsis pubescens* Besser (278 visits), *Leucanthemum vulgare* Lam. (191 visits), and *Erigeron annuus* (L.) Pers. (153 visits). Few plant species (*N* = 9) were exclusively visited by honeybees, while many species were exclusively visited by wild pollinators (*N* = 102), among which there were many umbellifers such as *Daucus carota* L., *Anthriscus sylvestris* (L.) Hoffm., and *Heracleum sphondylium* L. The most generalist plant species were *Ranunculus acris* L. (attracting 40 pollinator species), *Trifolium pratense* L. (attracting 39 pollinator species), and *E. annuus* (attracting 37 pollinator species). Flower corolla length ranged from 0.05 mm of open disc flowers to 33 mm of *Calystegia sepium* (L.) R. Br. (Table S2).

### Overall functional traits of plants and pollinators

**Fig. 2:**
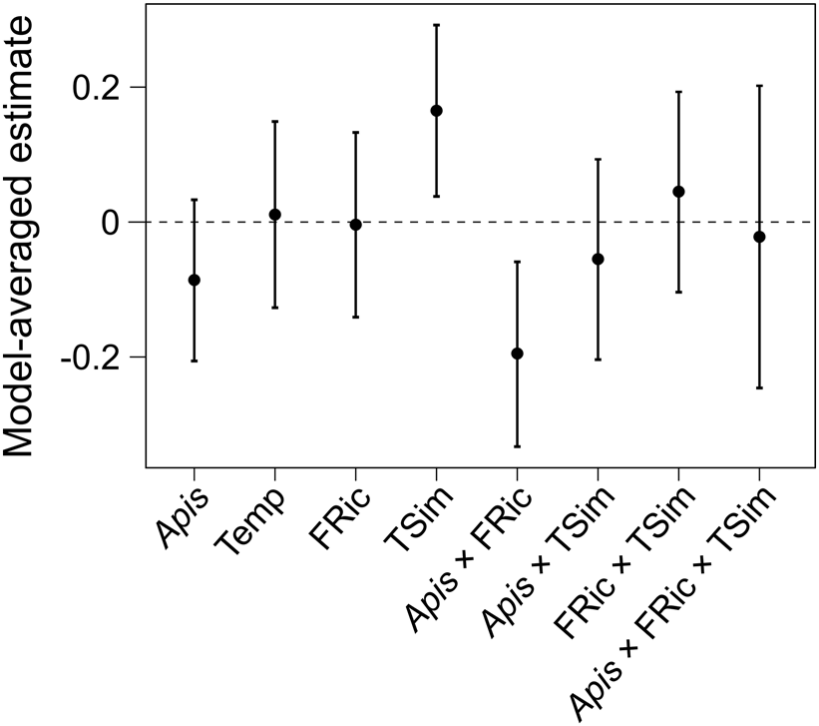
Model estimates from the model-averaging procedure based on the set of models with all functional traits of both plants and pollinators (Model 1). Explanatory variables of the global model are honeybee abundance (*Apis*, ln-transformed), temperature (Temp), standardized functional richness of plant community (FRic), trait similarity between wild pollinator community and the honeybee (TSim), and the interactions *Apis* × FRic, *Apis* × TSim, FRic × TSim, and *Apis* × FRic × TSim. All explanatory variables were scaled to mean 0 and standard deviation 1. Dots indicate the model estimated means, while error bars indicate the 95% confidence intervals for the expected values of the variables

**Fig. 3:**
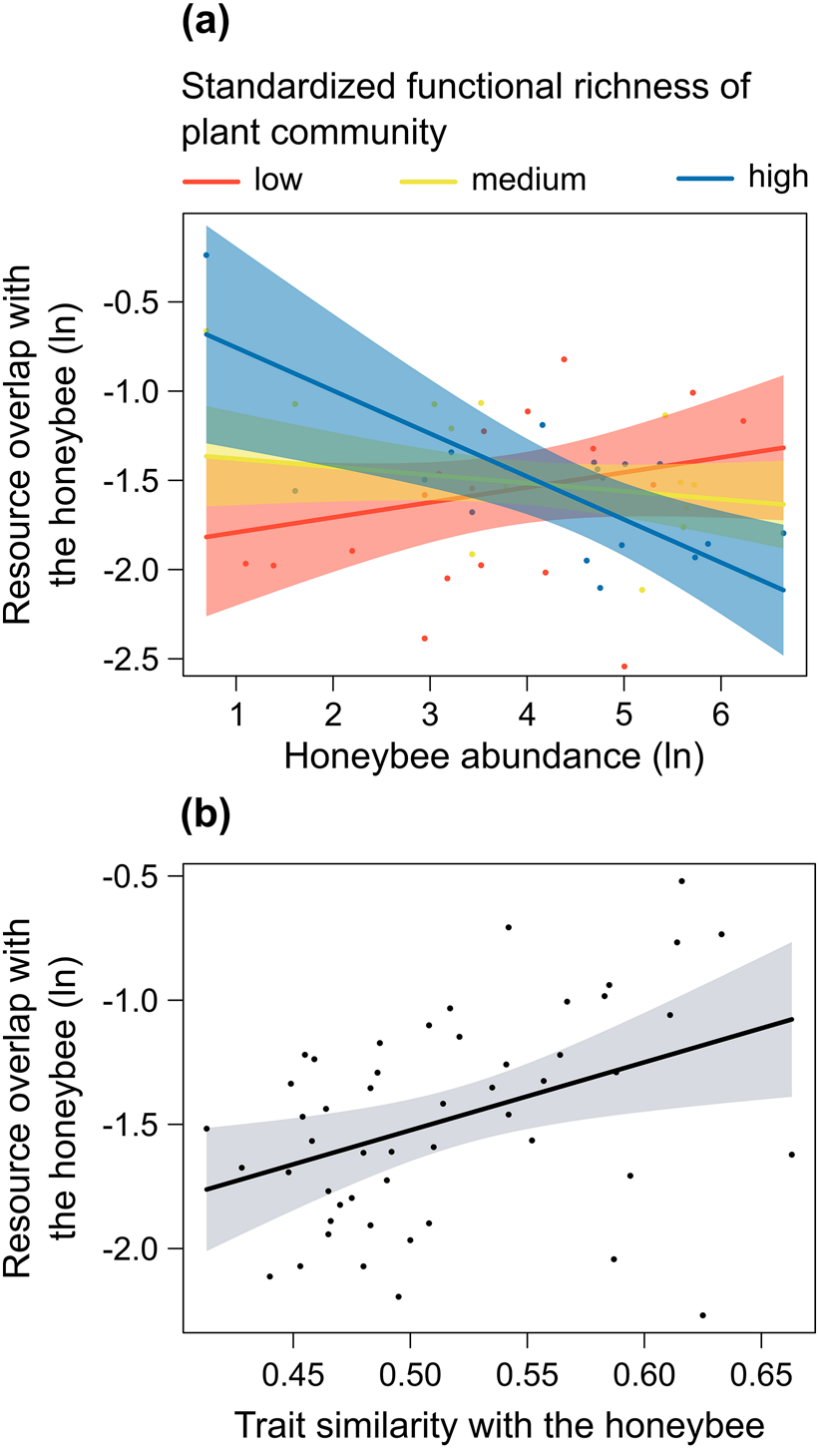
Partial residual plots showing the effect of **a** the interaction between honeybee abundance (ln-transformed) and standardized functional richness of plant community, with the three standardized functional richness levels representing the 10th, 50th, and 90th percentiles, and **b** trait similarity between wild pollinator community and the honeybee on resource overlap between wild pollinator community and the honeybee (ln-transformed) (Model 1). The shaded areas indicate the 95% confidence intervals for the expected values

For Model 1, fifteen models showed a ΔAICc < 6 (Table S6). Model averaging indicated that both plant and pollinator functional traits affected the resource overlap between wild pollinator community and the honeybee (Fig. 2). The impact of plant functional traits on resource overlap varied with honeybee abundance: resource overlap decreased as honeybee abundance increased in sites with high plant functional richness, while there was no change in resource overlap with increasing honeybee abundance in sites with low plant functional richness (Fig. 3a). Moreover, resource overlap increased as trait similarity between wild pollinator community and the honeybee increased (Fig. 3b). Temperature and other interactions did not affect the resource overlap (Table S6, Fig. 2).

For Model 2, twenty-eight models showed a ΔAICc < 6 (Table S7). The resource overlap was affected only by the trait similarity between wild pollinator community and the honeybee (Fig. S3).

### Single functional traits of pollinators

**Fig. 4:**
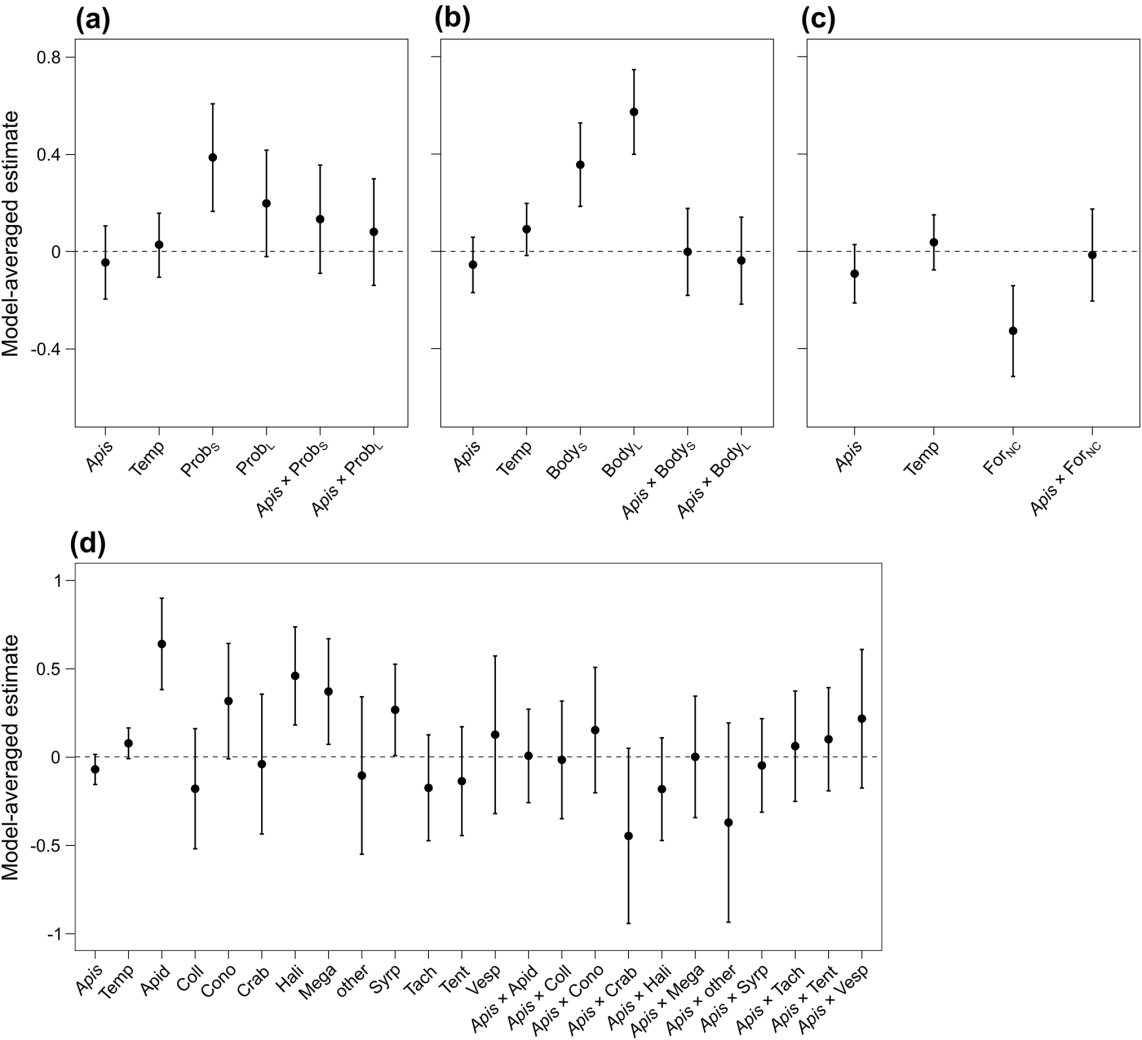
Model estimates from the model-averaging procedure based on the four sets of models considering single traits of pollinators, i.e., **a** proboscis length (Model 3), **b** body size (Model 4), **c** type of foraging range (Model 5), and **d** taxonomic family (Model 6). Explanatory variables of the four global models are honeybee abundance (*Apis*, ln-transformed), temperature (Temp), the levels of the four trait categories (*Prob*_*S*_ proboscis similar to the honeybee, *Prob*_*L*_ proboscis longer than the honeybee, *Body*_*S*_ body size similar to the honeybee, *Body*_*L*_ body size larger than the honeybee, *For*_*NC*_ non-central forager, *Apid* Apidae, *Coll* Colletidae, *Cono* Conopidae, *Crab* Crabronidae, *Hali* Halictidae, *Mega* Megachilidae, *other* other families, i.e., Cimbicidae, Megalodontesidae, Melittidae, and Scoliidae, *Syrp* Syrphidae, *Tach* Tachinidae, *Tent* Tenthredinidae, *Vesp* Vespidae) and the interactions between honeybee abundance and each levels of the traits. All continuous explanatory variables were scaled to mean 0 and standard deviation 1. Dots indicate the model estimated means, while error bars indicate the 95% confidence intervals for the expected values of the variables

**Fig. 5:**
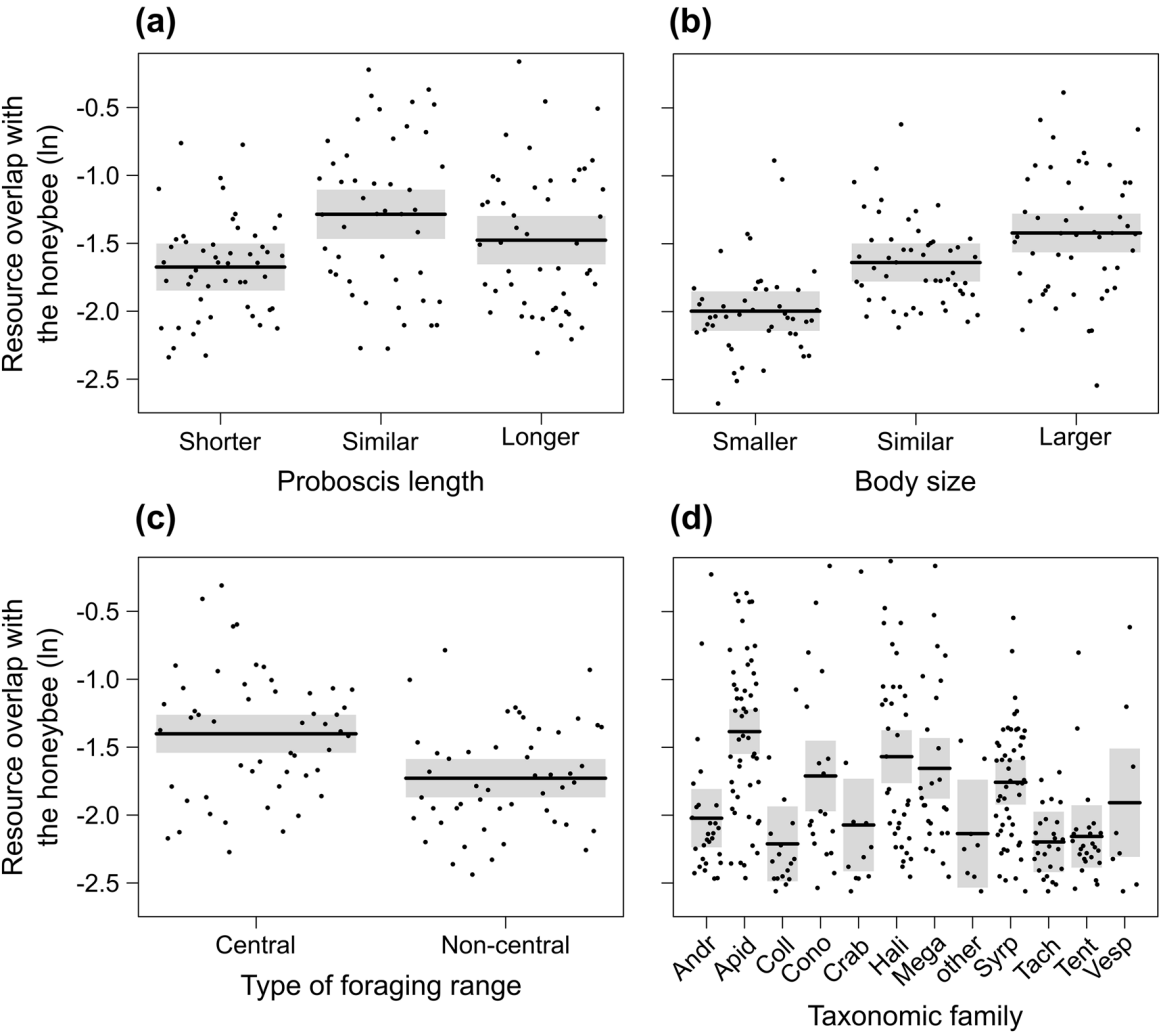
Partial residual plots showing the effect of **a** proboscis length (Model 3), **b** body size (Model 4), **c** type of foraging range (Model 5), and **d** taxonomic family (Model 6) on resource overlap between wild pollinator community and the honeybee (ln-transformed). The shaded areas indicate the 95% confidence intervals for the expected values

For Model 3, the multi-model inference analysis selected five models with a ΔAICc < 6 (Table S8a). Proboscis length was the only variable affecting the resource overlap between wild pollinator community and the honeybee (Fig. 4a), i.e., pollinators with proboscis length similar to the honeybee showed the highest overlap (Fig. 5a).

For Model 4, five models had a ΔAICc < 6 (Table S8b). Body size was the only variable affecting resource overlap between wild pollinator community and the honeybee (Fig. 4b), i.e., resource overlap increased with increasing body size (Fig. 5b). Models for body size showed the highest values of *R*^2^ compared to other functional traits (Table S8).

For Model 5, six models had a ΔAICc < 6 (Table S8c). Again, only the trait category strongly affected resource overlap between wild pollinator community and the honeybee (Fig. 4c), i.e., central-place foragers showed a higher overlap with honeybees compared to non-central-place foragers (Fig. 5c).

For Model 6, four models showed a ΔAICc < 6 (Table S8d). The taxonomic family strongly affected resource overlap between wild pollinator community and the honeybee (Fig. 4d). Bees of family Apidae showed a higher resource overlap than the other families (Fig. 5d), but the resource overlap was also relatively high for other families such as Conopidae, Halictidae, Megachilidae, and Syrphidae (Fig. 5d). We did not find an interactive effect of honeybee abundance and trait category in any of the models (Fig. 4), meaning that the difference in resource overlap between trait categories was independent of honeybee abundance.

## Discussion

Incorporating functional traits into ecological network analyses helped to elucidate the degree of resource overlap between wild pollinators and the honeybee. In particular, a low functional diversity of plant community combined with a high trait similarity between wild pollinators and the honeybee appeared to increase the risk of potential negative impacts of a high honeybee abundance on wild pollinator communities.

In areas with a high abundance of managed pollinators, resource overlap between wild pollinators and the honeybee could be mitigated by a high functional richness of plant community, in which pollinators could shift to alternative food resources, as opposed to areas with a low functional richness. To our knowledge, this is the first time that plant functional diversity was used to explore the changes in the resource overlap between wild pollinators and the honeybee. Previous works highlighted a similar effect of plant diversity and honeybee abundance on pollinator communities, with a reduction of potential competition in sites rich in plant species despite an increase in honeybee abundance (Rodríguez et al. 2021). Similarly, heterogeneous landscapes have been shown to support wild pollinators by reducing competition with honeybees (Herbertsson et al. 2016), while a lower availability of differentiated floral resources might increase competition among pollinator species (Thomson 2016; Wignall et al. 2020a, b). However, in contrast with previous research, we found that the resource overlap between wild pollinators and the honeybee never increased with increasing honeybee abundance (Lindström et al. 2016; but see Hudewenz and Klein 2015), even in sites with low plant functional diversity. This might be related to the honeybee foraging behaviour, as it often focuses on the most abundant and rewarding resources, especially in areas with low diversity of plants (Magrach et al. 2017). On the other hand, the lower resource overlap observed in sites with high functional diversity of plant community and high honeybee abundance could be related to the foraging behaviour of wild pollinators that could be forced to forage on plants that are not visited by honeybees. However, while we found an effect of functional richness of plant community, we observed no effect of functional dispersion. This could be partly explained by the fact that many sites were characterized by the same dominant plant species (e.g., *E. annuus* and *Melilotus albus* Medikus) and many different species with lower abundances, so functional dispersion values were similar across sites.

As expected, the resource overlap increased with increasing trait similarity between wild pollinators and the honeybee. Species with similar functional traits usually exploit similar floral resources (Fontaine et al. 2006; Albrecht et al. 2012), so potential competition is expected to be higher for wild pollinators which share traits with the honeybee. First, proboscis length is one of the main constraints of resource selection, affecting whether a pollinator species can obtain nectar from specific flowers. Pollinators are usually more efficient when foraging on plants with flower corolla length matching their mouthpart length (Inouye 1980; Madjidian et al. 2008; Klumpers et al. 2019). For example, hoverflies with a short proboscis tend to prefer flowers that are flat or have a shallow corolla (Fontaine et al. 2006), while long-tongued bumblebees tend to forage on flowers with deep corolla (Balfour et al. 2013). While pollinator species with proboscis shorter or longer than the honeybee mostly foraged on plant species that were not visited by honeybees, pollinators with a similar proboscis visited the same plant species, therefore, increasing their potential competition. Second, body size determines how far pollinators are able to forage, with large pollinators usually having a longer foraging range compared to small species (Gathmann and Tscharntke 2002; Greenleaf et al. 2007). Here, we found that body size was a key functional trait, driving the resource overlap between wild pollinators and the honeybee. The latter increased with increasing body size, even if we expected a higher overlap for species similar in size to the honeybee. Potential competition with honeybees was, therefore, higher for large species, such as bumblebees. Third, we also observed an increase in resource overlap for central-place foragers. These species are obliged to forage relatively near the nest, based on their foraging range, and are, therefore, unable to expand their foraging area, even when the local density of honeybees is high (Walther-Hellwig et al. 2006). Fourth, many Hymenoptera families such as Apidae, Halictidae, and Megachilidae showed a high level of resource overlap with honeybees. Surprisingly, both thick-headed flies (Diptera: Conopidae) and hoverflies (Diptera: Syrphidae), which we expected to mostly visit open disc flowers, also showed a relatively high resource overlap. While the potential negative effects of honeybees on wild pollinators have often focused on wild bees (e.g., Mallinger et al. 2017), other groups of insects might also be affected.

As the honeybee is not particularly active at low temperatures (Jaffé et al. 2010), we expected that its effect on wild pollinators would be stronger in sites with relatively high temperatures. However, similarly to what was observed in other works (e.g., Corcos et al. 2020; Seoane et al. 2021), we did not find any effect of temperature on resource overlap between wild pollinators and the honeybee, even if the observed temperature range was large (min = 18 °C, max = 38 °C).

## Conclusions

Honeybees have been introduced worldwide, and, therefore, often cohabit with wild pollinators. As their hives can host more than 50,000 individuals, their abundance in natural and managed habitats can be extremely high. Here, we showed that the potential interactions between wild pollinators and honeybees depended on functional traits of both plants and pollinators. In particular, our results highlight the potential role of plant functional diversity in supporting wild pollinators in areas with high honeybee density by decreasing the resource overlap between wild pollinators and the honeybee. Moreover, as pollinator species with traits similar to those of the honeybee tended to visit the same plant species, they could be more vulnerable to potential competition. From a conservation point of view, particular attention should be paid to the potential effects of beekeeping in sites where pollinator species of conservation concern possess functional traits similar to those of the honeybee. More research is needed to quantify potential short- and long-term effects of high honeybee abundance on fitness, health, and population dynamics of wild pollinators.

## Supplementary Information

Below is the link to the electronic supplementary material.

Supplementary file1 (DOCX 1932 KB)

## Data Availability

Once the paper will be accepted, the data supporting the results will be archived in a Zenodo Digital Repository.

## Code availability

Once the paper will be accepted, the R code will be archived in a Zenodo Digital Repository.
