# Supplementary material for: Functional traits of plants and pollinators explain resource overlap between honeybees and wild pollinators
Source: Oecologia. 2022 Apr 5;198(4):1019–29. doi: 10.1007/s00442-022-05151-6 (PMC9056470; doi:10.1007/s00442-022-05151-6)
Supplement: Supplementary file 1 — Supplementary file1 (DOCX 1932 KB) [file 442_2022_5151_MOESM1_ESM.docx]

**Table S1** Latitude, longitude, elevation (m a.s.l.), recorded air temperature (°C), number of flowering plant species (Plant rich), number of pollinator species (Poll rich), abundance of honeybees (Honeybee ab), and abundance of wild pollinators (Wild poll ab) for each sampling site

| **Site code** | **Latitude (WGS84)** | **Longitude (WGS84)** | **Elevation (m a.s.l.)** | **Temperature (°C)** | **Plant rich** | **Poll rich** | **Honeybee ab** | **Wild poll ab** |
| --- | --- | --- | --- | --- | --- | --- | --- | --- |
| A | 45.587007 | 11.468313 | 60 | 27.67 | 20 | 31 | 5 | 111 |
| AA | 46.487789 | 12.035109 | 2005 | 25.29 | 26 | 22 | 768 | 73 |
| AB | 46.490416 | 12.066126 | 1982 | 23.24 | 28 | 12 | 109 | 16 |
| AC | 46.518712 | 12.007109 | 2090 | 22.36 | 34 | 19 | 145 | 39 |
| AD | 46.533594 | 11.986739 | 2111 | 27.05 | 25 | 13 | 282 | 43 |
| AE | 46.565153 | 12.243253 | 1658 | 22.37 | 21 | 30 | 34 | 65 |
| AF | 46.548764 | 12.261756 | 1500 | 26.72 | 19 | 16 | 22 | 29 |
| AG | 46.396688 | 12.104436 | 1450 | 25.43 | 27 | 49 | 274 | 119 |
| AH | 46.420795 | 12.105355 | 1780 | 20.95 | 28 | 30 | 31 | 150 |
| AI | 46.484398 | 11.831885 | 2008 | 19.63 | 22 | 14 | 2 | 93 |
| AJ | 45.82542 | 12.180076 | 175 | 27.99 | 13 | 27 | 44 | 63 |
| AK | 45.808239 | 12.111145 | 320 | 30.23 | 11 | 14 | 80 | 25 |
| AL | 46.485461 | 11.788397 | 1915 | 19.52 | 34 | 34 | 101 | 110 |
| AM | 46.549767 | 11.813354 | 2055 | 19.02 | 35 | 30 | 116 | 78 |
| AN | 46.339973 | 11.804519 | 2032 | 20.95 | 13 | 19 | 9 | 130 |
| AO | 46.195026 | 11.42058 | 1430 | 22.72 | 18 | 26 | 34 | 51 |
| AP | 46.348829 | 11.845274 | 1600 | 22.33 | 18 | 16 | 25 | 61 |
| AQ | 46.139193 | 11.472058 | 1250 | 24.49 | 16 | 24 | 149 | 332 |
| AR | 46.147208 | 11.771577 | 910 | 30.98 | 15 | 17 | 4 | 31 |
| AS | 46.172563 | 11.441852 | 2040 | 19.65 | 16 | 22 | 227 | 64 |
| AT | 45.880301 | 11.794101 | 1665 | 21.37 | 18 | 24 | 2 | 75 |
| AU | 45.755492 | 10.874539 | 1500 | 18.05 | 11 | 10 | 3 | 25 |
| AV | 45.756764 | 10.920759 | 640 | 24.63 | 10 | 9 | 108 | 29 |
| AW | 45.803576 | 12.049459 | 200 | 35.54 | 8 | 28 | 201 | 67 |
| AX | 45.868023 | 12.009987 | 170 | 30.91 | 10 | 13 | 24 | 45 |
| AZ | 45.694425 | 10.926014 | 130 | 32.84 | 10 | 12 | 55 | 16 |
| B | 45.583447 | 11.463828 | 150 | 27.37 | 22 | 29 | 25 | 119 |
| C | 45.747647 | 11.329589 | 670 | 24.04 | 19 | 35 | 113 | 109 |
| D | 45.755546 | 11.363854 | 630 | 24.65 | 17 | 29 | 64 | 69 |
| E | 45.711296 | 11.68899 | 76 | 30.60 | 15 | 32 | 302 | 94 |
| F | 45.760339 | 11.699603 | 106 | 36.89 | 12 | 25 | 508 | 73 |
| G | 45.595876 | 11.467499 | 150 | 37.89 | 22 | 22 | 19 | 121 |
| H | 45.622053 | 11.415189 | 165 | 33.93 | 16 | 19 | 19 | 103 |
| I | 45.862562 | 11.762562 | 1251 | 21.02 | 19 | 27 | 353 | 57 |
| J | 45.649502 | 11.7196 | 50 | 28.19 | 11 | 9 | 31 | 25 |
| K | 45.979623 | 12.730447 | 35 | 27.88 | 20 | 25 | 179 | 72 |
| L | 45.839134 | 11.736687 | 1000 | 25.27 | 15 | 17 | 550 | 51 |
| M | 45.848196 | 11.452165 | 943 | 26.37 | 29 | 29 | 119 | 93 |
| N | 45.824215 | 11.455581 | 1170 | 23.79 | 19 | 18 | 31 | 71 |
| O | 45.760665 | 11.39284 | 1215 | 19.51 | 22 | 34 | 21 | 119 |
| P | 46.137372 | 11.502687 | 1170 | 23.54 | 20 | 20 | 44 | 114 |
| Q | 46.121675 | 11.49488 | 920 | 27.36 | 19 | 22 | 66 | 61 |
| R | 46.154322 | 11.424568 | 1530 | 25.61 | 17 | 22 | 19 | 36 |
| S | 46.068526 | 11.431498 | 870 | 26.67 | 24 | 29 | 306 | 77 |
| T | 46.006465 | 11.404734 | 890 | 28.29 | 22 | 16 | 266 | 60 |
| U | 46.012846 | 11.432452 | 825 | 29.46 | 31 | 21 | 215 | 41 |
| V | 45.670237 | 11.056824 | 1430 | 24.46 | 32 | 35 | 5 | 191 |
| W | 46.196241 | 12.658885 | 870 | 21.16 | 25 | 29 | 150 | 112 |
| X | 45.758992 | 11.192283 | 1000 | 23.27 | 30 | 38 | 309 | 143 |
| Y | 45.769546 | 11.136029 | 860 | 24.26 | 20 | 34 | 115 | 109 |
| Z | 45.637685 | 11.720541 | 40 | 30.60 | 16 | 22 | 35 | 63 |

**Table S2** Functional traits of flowering plant species: flower colour (six classes: blue, brown, pink/purple, red, white, yellow/orange), flower type (nine classes: bell/funnel/lip, brush, hidden disc, open disc, flag, flower head, NA, pollen, and stalk disc flower), and flower corolla length in mm

| **Plant species** | **Flower color** | **Flower type** | **Corolla (mm)** |
| --- | --- | --- | --- |
| *Achillea clavennae* | white | flower head | 6 |
| *Achillea millefolium* | white | flower head | 4.7 |
| *Aconitum degenii* | blue | bell/funnel/lip | 26 |
| *Aconitum lycoctonum* | yellow/orange | bell/funnel/lip | 25.5 |
| *Aconitum napellus* | blue | bell/funnel/lip | 11.5 |
| *Adenostyles alpina* | pink/purple | stalk disc | 13 |
| *Aegopodium podagraria* | white | open disc | 1 |
| *Agrimonia eupatoria* | yellow/orange | pollen | 0.05 |
| *Allium carinatum* | pink/purple | hidden disc | 5.5 |
| *Allium schoenoprasum* | pink/purple | hidden disc | 12 |
| *Angelica sylvestris* | white | open disc | 1 |
| *Anthriscus sylvestris* | white | open disc | 0.4 |
| *Anthyllis vulneraria* | yellow/orange | flag | 12 |
| *Aristolochia clematitis* | yellow/orange | bell/funnel/lip | 30 |
| *Arnica montana* | yellow/orange | flower head | 17.5 |
| *Asperula cristata* | pink/purple | bell/funnel/lip | 3 |
| *Aster alpinus* | pink/purple | flower head | 8 |
| *Astragalus glycyphyllos* | yellow/orange | flag | 7 |
| *Astrantia major* | white | flower head | 6 |
| *Bellis perennis* | white | flower head | 4 |
| *Betonica alopecurus* | yellow/orange | bell/funnel/lip | 10 |
| *Buddleja davidii* | pink/purple | stalk disc | 10 |
| *Buphthalmum salicifolium* | yellow/orange | flower head | 8 |
| *Calluna vulgaris* | pink/purple | bell/funnel/lip | 5 |
| *Calystegia sepium* | white | bell/funnel/lip | 33.3 |
| *Campanula barbata* | pink/purple | bell/funnel/lip | 16 |
| *Campanula carnica* | pink/purple | bell/funnel/lip | 11 |
| *Campanula glomerata* | pink/purple | bell/funnel/lip | 13 |
| *Campanula patula* | pink/purple | bell/funnel/lip | 11 |
| *Campanula persicifolia* | pink/purple | bell/funnel/lip | 18 |
| *Campanula rapunculoides* | pink/purple | bell/funnel/lip | 17 |
| *Campanula rapunculus* | pink/purple | bell/funnel/lip | 15 |
| *Campanula rotundifolia* | pink/purple | bell/funnel/lip | 15 |
| *Campanula scheuchzeri* | pink/purple | bell/funnel/lip | 15 |
| *Campanula spicata* | pink/purple | bell/funnel/lip | 11 |
| *Campanula trachelium* | pink/purple | bell/funnel/lip | 26.5 |
| *Carduus defloratus* | pink/purple | flower head | 17 |
| *Carduus nutans* | pink/purple | flower head | 12 |
| *Carduus personata* | pink/purple | flower head | 22 |
| *Carum carvi* | white | open disc | 0.3 |
| *Centaurea jacea* | pink/purple | flower head | 20 |
| *Centaurea nervosa* | pink/purple | flower head | 23.5 |
| *Centaurea nigrescens* | pink/purple | flower head | 12.2 |
| *Centaurea scabiosa* | pink/purple | flower head | 13.5 |
| *Centaurea stoebe* | pink/purple | flower head | 10 |
| *Centaurea triumfetti* | blue | flower head | 12 |
| *Cerastium arvense* | white | hidden disc | 4 |
| *Cerastium holosteoides* | white | hidden disc | 2 |
| *Chaerophyllum hirsutum* | white | open disc | 1 |
| *Cichorium intybus* | blue | flower head | 0.05 |
| *Cirsium arvense* | pink/purple | flower head | 16 |
| *Cirsium erisithales* | yellow/orange | flower head | 23.3 |
| *Cirsium heterophyllum* | pink/purple | flower head | 33 |
| *Cirsium montanum* | pink/purple | flower head | 7 |
| *Cirsium oleraceum* | yellow/orange | flower head | 23 |
| *Clematis vitalba* | white | pollen | 0.05 |
| *Clinopodium acinos* | pink/purple | bell/funnel/lip | 9 |
| *Clinopodium alpinum* | pink/purple | bell/funnel/lip | 9 |
| *Clinopodium nepeta* | pink/purple | bell/funnel/lip | 9.5 |
| *Clinopodium vulgare* | pink/purple | bell/funnel/lip | 11 |
| *Convolvulus arvensis* | white | bell/funnel/lip | 16.3 |
| *Conyza canadensis* | white | flower head | 5 |
| *Crepis aurea* | yellow/orange | flower head | 13.5 |
| *Crepis biennis* | yellow/orange | flower head | 8.5 |
| *Crepis foetida* | yellow/orange | flower head | 6 |
| *Crepis paludosa* | yellow/orange | flower head | 11 |
| *Crepis vesicaria* | yellow/orange | flower head | 8 |
| *Cruciata laevipes* | yellow/orange | open disc | 0.05 |
| *Daucus carota* | white | open disc | 0.05 |
| *Delosperma* sp. | pink/purple | flower head | 5 |
| *Dianthus superbus* | pink/purple | stalk disc | 30 |
| *Diplotaxis tenuifolia* | yellow/orange | hidden disc | 4 |
| *Doronicum austriacum* | yellow/orange | flower head | 4.5 |
| *Dorycnium pentaphyllum* | white | flag | 4 |
| *Dryas octopetala* | white | hidden disc | 0.05 |
| *Echium vulgare* | pink/purple | bell/funnel/lip | 9.3 |
| *Epilobium angustifolium* | pink/purple | hidden disc | 0.05 |
| *Epilobium dodonaei* | pink/purple | hidden disc | 0.05 |
| *Epilobium hirsutum* | pink/purple | hidden disc | 0.05 |
| *Epilobium montanum* | pink/purple | hidden disc | 7 |
| *Erigeron annuus* | white | flower head | 1 |
| *Eupatorium cannabinum* | pink/purple | flower head | 5 |
| *Euphrasia rostkoviana* | white | bell/funnel/lip | 10 |
| *Euphrasia salisburgensis* | white | bell/funnel/lip | 6 |
| *Euphrasia* sp. | white | bell/funnel/lip | 6.5 |
| *Filipendula vulgaris* | white | pollen | 0.05 |
| *Fragaria vesca* | white | hidden disc | 0.05 |
| *Galeopsis pubescens* | pink/purple | bell/funnel/lip | 17 |
| *Galeopsis speciosa* | yellow/orange | bell/funnel/lip | 22 |
| *Galeopsis tetrahit* | pink/purple | bell/funnel/lip | 13 |
| *Galium lucidum* | white | open disc | 0.5 |
| *Galium mollugo* | white | open disc | 1 |
| *Galium saxatile* | white | open disc | 1 |
| *Galium verum* | yellow/orange | open disc | 0.3 |
| *Genista tinctoria* | yellow/orange | flag | 7.7 |
| *Gentiana cruciata* | blue | bell/funnel/lip | 14.5 |
| *Gentianella rhaetica* | pink/purple | bell/funnel/lip | 17 |
| *Geranium columbinum* | pink/purple | hidden disc | 0.05 |
| *Geranium molle* | pink/purple | hidden disc | 0.05 |
| *Geranium phaeum* | pink/purple | hidden disc | 0.05 |
| *Geranium pyrenaicum* | pink/purple | hidden disc | 0.05 |
| *Geranium robertianum* | pink/purple | hidden disc | 6.5 |
| *Geranium sylvaticum* | pink/purple | hidden disc | 0.05 |
| *Geum rivale* | red | bell/funnel/lip | 7 |
| *Gymandenia conopsea* | pink/purple | bell/funnel/lip | 6 |
| *Gypsophila repens* | white | bell/funnel/lip | 7 |
| *Hedysarum hedysaroides* | pink/purple | flag | 8 |
| *Helianthemum nummularium* | yellow/orange | pollen | 0.05 |
| *Heracleum sphondylium* | white | open disc | 0.1 |
| *Hieracium bifidum* | yellow/orange | flower head | 13.3 |
| *Hieracium glaucum* | yellow/orange | flower head | 12 |
| *Hieracium picroides* | yellow/orange | flower head | 11 |
| *Hieracium pilosella* | yellow/orange | flower head | 11 |
| *Hieracium* sp. | yellow/orange | flower head | 8.5 |
| *Hieracium valdepilosum* | yellow/orange | flower head | 20 |
| *Horminum pyrenaicum* | pink/purple | bell/funnel/lip | 14 |
| *Hypericum maculatum* | yellow/orange | pollen | 0.05 |
| *Hypericum perforatum* | yellow/orange | pollen | 0.05 |
| *Hypochaeris uniflora* | yellow/orange | flower head | 22 |
| *Impatiens glandulifera* | pink/purple | bell/funnel/lip | 23 |
| *Inula salicina* | yellow/orange | flower head | 7.5 |
| *Inula* sp. | yellow/orange | flower head | 9 |
| *Jacobaea alpina* | yellow/orange | flower head | 7.5 |
| *Knautia arvensis* | blue | flower head | 7.3 |
| *Knautia drymeia* | pink/purple | flower head | 7 |
| *Knautia longifolia* | pink/purple | flower head | 9 |
| *Lamium album* | white | bell/funnel/lip | 9.5 |
| *Lamium galeobdolon* | yellow/orange | bell/funnel/lip | 9 |
| *Lamium orvala* | pink/purple | bell/funnel/lip | 14 |
| *Lathyrus pratensis* | yellow/orange | flag | 13.7 |
| *Lathyrus sylvestris* | pink/purple | flag | 12.5 |
| *Leontodon hispidus* | yellow/orange | flower head | 11.7 |
| *Leucanthemum vulgare* | white | flower head | 4.8 |
| *Ligustrum lucidum* | white | bell/funnel/lip | 3 |
| *Ligustrum vulgare* | white | bell/funnel/lip | 3 |
| *Lilium bulbiferum* | yellow/orange | bell/funnel/lip | 0.05 |
| *Lilium martagon* | pink/purple | bell/funnel/lip | 10 |
| *Linaria alpina* | pink/purple | bell/funnel/lip | 8 |
| *Loncomelos brevistylus* | white | hidden disc | 0.05 |
| *Lotus corniculatus* | yellow/orange | flag | 5.5 |
| *Lupinus polyphyllus* | blue | flag | 9 |
| *Lychnis flos cuculi* | pink/purple | stalk disc | 10.5 |
| *Lysimachia arvensis* | yellow/orange | pollen | 0.05 |
| *Lysimachia vulgaris* | yellow/orange | pollen | 0.05 |
| *Lythrum salicaria* | pink/purple | bell/funnel/lip | 7 |
| *Malva sylvestris* | pink/purple | hidden disc | 0.05 |
| *Matricaria chamomilla* | white | flower head | 7 |
| *Medicago falcata* | yellow/orange | flag | 5 |
| *Medicago lupulina* | yellow/orange | flag | 2.5 |
| *Medicago sativa* | pink/purple | flag | 6 |
| *Melampyrum italicum* | yellow/orange | bell/funnel/lip | 18 |
| *Melampyrum* sp. | yellow/orange | bell/funnel/lip | 10 |
| *Melilotus albus* | white | flag | 3.3 |
| *Mentha arvensis* | pink/purple | bell/funnel/lip | 4 |
| *Mentha longifolia* | pink/purple | bell/funnel/lip | 3 |
| *Minuartia recurva* | white | hidden disc | 0.05 |
| *Myosotis* sp. | blue | stalk disc | 2.3 |
| *Myosoton aquaticum* | white | hidden disc | 0.05 |
| *Oenothera biennis* | yellow/orange | stalk disc | 17 |
| *Onobrychis montana* | pink/purple | flag | 11.7 |
| *Onobrychis viciifolia* | pink/purple | flag | 11.5 |
| *Ononis spinosa* | pink/purple | flag | 10 |
| *Origanum vulgare* | pink/purple | bell/funnel/lip | 6 |
| *Ornithogalum pyrenaicum* | white | hidden disc | 0.05 |
| *Ornithogalum umbellatum* | white | hidden disc | 1 |
| *Oxalis articulata* | pink/purple | hidden disc | 3 |
| *Oxytropis montana* | pink/purple | flag | 11.5 |
| *Papaver rhoeas* | red | pollen | 0.05 |
| *Parnassia palustris* | white | open disc | 0.05 |
| *Pastinaca sativa* | yellow/orange | open disc | 1 |
| *Pedicularis comosa* | yellow/orange | bell/funnel/lip | 17 |
| *Pedicularis palustris* | pink/purple | bell/funnel/lip | 14 |
| *Persicaria bistorta* | pink/purple | bell/funnel/lip | 2 |
| *Petrorhagia saxifraga* | pink/purple | bell/funnel/lip | 4.7 |
| *Phyteuma betonicifolium* | blue | NA | 4.8 |
| *Phyteuma orbiculare* | blue | NA | 4 |
| *Picris hieracioides* | yellow/orange | flower head | 12.3 |
| *Pimpinella major* | white | open disc | 1.3 |
| *Pimpinella saxifraga* | white | open disc | 0.05 |
| *Plantago lanceolata* | white | NA | 0.05 |
| *Plantago media* | white | NA | 0.05 |
| *Polygonum viviparum* | white | bell/funnel/lip | 2 |
| *Potentilla argentea* | yellow/orange | hidden disc | 0.05 |
| *Potentilla erecta* | yellow/orange | hidden disc | 0.05 |
| *Potentilla nitida* | pink/purple | hidden disc | 0.05 |
| *Potentilla reptans* | yellow/orange | hidden disc | 0.05 |
| *Prenanthes purpurea* | pink/purple | flower head | 11.5 |
| *Prunella grandiflora* | pink/purple | bell/funnel/lip | 13 |
| *Prunella laciniata* | white | bell/funnel/lip | 6 |
| *Prunella vulgaris* | pink/purple | bell/funnel/lip | 8.4 |
| *Pyrola minor* | white | bell/funnel/lip | 0.05 |
| *Ranunculus acris* | yellow/orange | hidden disc | 0.05 |
| *Ranunculus platanifolius* | white | hidden disc | 0.05 |
| *Ranunculus polyanthemophyllus* | yellow/orange | hidden disc | 0.05 |
| *Ranunculus repens* | yellow/orange | hidden disc | 0.05 |
| *Rhinanthus alectorolophus* | yellow/orange | bell/funnel/lip | 22.5 |
| *Rhinanthus freynii* | yellow/orange | bell/funnel/lip | 18 |
| *Rhinanthus minor* | yellow/orange | bell/funnel/lip | 16 |
| *Rhododendron hirsutum* | pink/purple | bell/funnel/lip | 10.5 |
| *Rorippa sylvestris* | yellow/orange | hidden disc | 2 |
| *Rosa canina* | pink/purple | pollen | 0.05 |
| *Rubus* sp. | white | hidden disc | 0.05 |
| *Salvia glutinosa* | yellow/orange | bell/funnel/lip | 20 |
| *Salvia pratensis* | blue | bell/funnel/lip | 9 |
| *Saponaria officinalis* | pink/purple | stalk disc | 23 |
| *Saxifraga rotundifolia* | white | open disc | 0.05 |
| *Scabiosa columbaria* | pink/purple | flower head | 5.5 |
| *Scabiosa gramuntia* | pink/purple | flower head | 6.5 |
| *Scabiosa lucida* | pink/purple | flower head | 6 |
| *Scabiosa triandra* | pink/purple | flower head | 6.4 |
| *Scrophularia nodosa* | red | bell/funnel/lip | 6 |
| *Securigera varia* | pink/purple | flag | 11 |
| *Sedum acre* | yellow/orange | hidden disc | 1.75 |
| *Sedum album* | white | hidden disc | 1.5 |
| *Senecio inaequidens* | yellow/orange | flower head | 8 |
| *Senecio nemorensis* | yellow/orange | flower head | 13 |
| *Senecio squalidus* | yellow/orange | flower head | 10.3 |
| *Sherardia arvensis* | pink/purple | bell/funnel/lip | 2.5 |
| *Silene alba* | white | stalk disc | 16 |
| *Silene dioica* | pink/purple | stalk disc | 15.3 |
| *Silene nutans* | white | stalk disc | 13 |
| *Silene saxifraga* | white | stalk disc | 5.5 |
| *Silene vulgaris* | white | stalk disc | 18 |
| *Solanum tuberosum* | white | pollen | 2 |
| *Solidago gigantea* | yellow/orange | flower head | 6 |
| *Solidago virgaurea* | yellow/orange | flower head | 10.7 |
| *Stachys alpina* | pink/purple | bell/funnel/lip | 10 |
| *Stachys officinalis* | pink/purple | bell/funnel/lip | 9 |
| *Stachys recta* | white | bell/funnel/lip | 8 |
| *Stachys sylvatica* | pink/purple | bell/funnel/lip | 11 |
| *Stellaria graminea* | white | hidden disc | 0.05 |
| *Stellaria nemorum* | white | hidden disc | 2.5 |
| *Symphytum officinale* | pink/purple | bell/funnel/lip | 15 |
| *Tanacetum corymbosum* | white | flower head | 8 |
| *Tanacetum vulgare* | yellow/orange | flower head | 5 |
| *Taraxacum officinale* | yellow/orange | flower head | 8 |
| *Teucrium chamaedrys* | pink/purple | bell/funnel/lip | 8 |
| *Thalictrum aquilegifolium* | pink/purple | brush | 0.05 |
| *Thalictrum lucidum* | yellow/orange | brush | 0.05 |
| *Thymus* sp. | pink/purple | bell/funnel/lip | 3.5 |
| *Torilis arvensis* | white | open disc | 0.1 |
| *Tragopogon pratensis* | yellow/orange | flower head | 12.5 |
| *Trifolium badium* | yellow/orange | flag | 5.5 |
| *Trifolium medium* | pink/purple | flag | 13 |
| *Trifolium montanum* | white | flag | 8.5 |
| *Trifolium pratense* | pink/purple | flag | 11.3 |
| *Trifolium repens* | white | flag | 4 |
| *Valeriana montana* | pink/purple | bell/funnel/lip | 5 |
| *Valeriana officinalis* | pink/purple | bell/funnel/lip | 3.7 |
| *Verbascum alpinum* | yellow/orange | bell/funnel/lip | 8 |
| *Verbascum chaixii* | yellow/orange | bell/funnel/lip | 5 |
| *Verbascum densiflorum* | yellow/orange | bell/funnel/lip | 10 |
| *Verbascum lychnitis* | yellow/orange | bell/funnel/lip | 10 |
| *Verbascum nigrum* | yellow/orange | bell/funnel/lip | 3 |
| *Verbena officinalis* | pink/purple | bell/funnel/lip | 5.3 |
| *Veronica anagallis-aquatica* | pink/purple | bell/funnel/lip | 0.05 |
| *Veronica chamaedrys* | blue | bell/funnel/lip | 0.05 |
| *Veronica persica* | blue | bell/funnel/lip | 0.05 |
| *Vicia cracca* | pink/purple | flag | 7.6 |
| *Vicia sepium* | pink/purple | flag | 10 |
| *Vicia sylvatica* | white | flag | 13 |
| *Vicia villosa* | pink/purple | flag | 11 |

**Table S3** Traits of pollinator species: taxonomic family, proboscis length in mm (mean value of the measured specimens), body size in mm (mean value of the measured specimens), and type of foraging range (*C* central-place forager, *NC* non-central-place forager)

| **Pollinator species** | **Family** | **Proboscis (mm)** | **Body (mm)** | **Foraging** |
| --- | --- | --- | --- | --- |
| *Abia fasciata* | Cimbicidae | 0.8 | 12 | NC |
| *Amegilla quadrifasciata* | Apidae | 8.2 | 13 | C |
| *Andrena aeneiventris* | Andrenidae | 1.1 | 7 | C |
| *Andrena alfkenella* | Andrenidae | 0.95 | 5.75 | C |
| *Andrena cineraria* | Andrenidae | 2 | 12 | C |
| *Andrena haemorrhoa* | Andrenidae | 1.6 | 9 | C |
| *Andrena hattorfiana* | Andrenidae | 3.5 | 14 | C |
| *Andrena intermedia* | Andrenidae | 2.3 | 11 | C |
| *Andrena labialis* | Andrenidae | 2 | 11.5 | C |
| *Andrena lathyri* | Andrenidae | 2.7 | 10 | C |
| *Andrena limata* | Andrenidae | 1.85 | 11 | C |
| *Andrena pandellei* | Andrenidae | 1.95 | 9.5 | C |
| *Andrena schencki* | Andrenidae | 2.8 | 12 | C |
| *Andrena* sp. 1 | Andrenidae | 1.6 | 9 | C |
| *Andrena* sp. 2 | Andrenidae | 1.45 | 9 | C |
| *Andrena* sp. 3 | Andrenidae | 1.5 | 8 | C |
| *Andrena* sp. 4 | Andrenidae | 1.03 | 6.75 | C |
| *Andrena* sp. 5 | Andrenidae | 1.15 | 10 | C |
| *Andrena* sp. 6 | Andrenidae | 1.2 | 10 | C |
| *Andrena* sp. 7 | Andrenidae | 1.25 | 8 | C |
| *Andrena subopaca* | Andrenidae | 1 | 5.5 | C |
| *Andrena wilkella* | Andrenidae | 1.95 | 9.5 | C |
| *Anthidiellum strigatum* | Megachilidae | 3.2 | 7 | C |
| *Anthidium florentinum* | Megachilidae | 4 | 13 | C |
| *Anthidium oblongatum* | Megachilidae | 2.7 | 7 | C |
| *Anthidium punctatum* | Megachilidae | 3.3 | 8 | C |
| *Anthophora balneorum* | Apidae | 12 | 15 | C |
| *Anthophora furcata* | Apidae | 8.25 | 11 | C |
| *Anthophora plumipes* | Apidae | 9 | 14 | C |
| *Anthophora salviae* | Apidae | 6 | 12 | C |
| *Apis mellifera* | Apidae | 5 | 12 | C |
| *Argogorytes mystaceus* | Crabronidae | 1.1 | 13 | C |
| *Athalia rosae* | Tenthredinidae | 0.5 | 6.5 | NC |
| *Billaea triangulifera* | Tachinidae | 2.3 | 10 | NC |
| *Bombus argillaceus* | Apidae | 10.7 | 15.67 | C |
| *Bombus barbutellus* | Apidae | 7.15 | 17 | NC |
| *Bombus bohemicus* | Apidae | 6.35 | 17.75 | NC |
| *Bombus campestris* | Apidae | 6.2 | 15 | NC |
| *Bombus gerstaeckeri* | Apidae | 16 | 18 | C |
| *Bombus hortorum* | Apidae | 12.53 | 16.83 | C |
| *Bombus humilis* | Apidae | 8.33 | 15 | C |
| *Bombus hypnorum* | Apidae | 7.25 | 13.5 | C |
| *Bombus inexspectatus* | Apidae | 7 | 15 | NC |
| *Bombus lapidarius* | Apidae | 7.27 | 15.33 | C |
| *Bombus lucorum* | Apidae | 5.5 | 16 | C |
| *Bombus mendax* | Apidae | 9.5 | 16 | C |
| *Bombus mesomelas* | Apidae | 8.5 | 15.5 | C |
| *Bombus monticola* | Apidae | 5.5 | 13 | C |
| *Bombus pascuorum* | Apidae | 8.25 | 15 | C |
| *Bombus pratorum* | Apidae | 7.67 | 14.67 | C |
| *Bombus pyrenaeus* | Apidae | 5.13 | 13.33 | C |
| *Bombus ruderarius* | Apidae | 7.13 | 13.25 | C |
| *Bombus rupestris* | Apidae | 7.4 | 18 | NC |
| *Bombus sichelii* | Apidae | 6.75 | 15.5 | C |
| *Bombus soroeensis* | Apidae | 6.35 | 17 | C |
| *Bombus sylvarum* | Apidae | 8.8 | 16 | C |
| *Bombus sylvestris* | Apidae | 6.5 | 16 | NC |
| *Bombus terrestris* | Apidae | 6.5 | 14.67 | C |
| *Bombus vestalis* | Apidae | 8.4 | 22 | NC |
| *Bombus wurflenii* | Apidae | 7.75 | 17 | C |
| *Callicera aurata* | Syrphidae | 1.5 | 12 | NC |
| *Ceratina chalybaea* | Apidae | 3.5 | 9.5 | C |
| *Ceratina cucurbitina* | Apidae | 3.5 | 8.5 | C |
| *Ceratina cyanea* | Apidae | 2.5 | 7.5 | C |
| *Ceratina dallatorreana* | Apidae | 2.6 | 6 | C |
| *Ceratina gravidula* | Apidae | 4.7 | 11 | C |
| *Cerceris rubida* | Crabronidae | 1.6 | 7.5 | C |
| *Cerceris sabulosa* | Crabronidae | 1.6 | 9 | C |
| *Cheilosia aerea* | Syrphidae | 1 | 8 | NC |
| *Cheilosia albipila* | Syrphidae | 1 | 10 | NC |
| *Cheilosia antiqua* | Syrphidae | 1 | 7 | NC |
| *Cheilosia canicularis* | Syrphidae | 1 | 12 | NC |
| *Cheilosia frontalis* | Syrphidae | 1 | 8.5 | NC |
| *Cheilosia illustrata* | Syrphidae | 1 | 10 | NC |
| *Cheilosia laticornis* | Syrphidae | 1 | 9 | NC |
| *Cheilosia latifrons* | Syrphidae | 1 | 9 | NC |
| *Cheilosia longula* | Syrphidae | 1 | 7.5 | NC |
| *Cheilosia mutabilis* | Syrphidae | 1 | 6.5 | NC |
| *Cheilosia nigripes* | Syrphidae | 1 | 7 | NC |
| *Cheilosia pagana* | Syrphidae | 1 | 6.5 | NC |
| *Cheilosia personata* | Syrphidae | 1 | 9.5 | NC |
| *Cheilosia proxima* | Syrphidae | 1 | 8 | NC |
| *Cheilosia ranunculi* | Syrphidae | 1 | 8 | NC |
| *Cheilosia scutellata* | Syrphidae | 1 | 8.5 | NC |
| *Cheilosia soror* | Syrphidae | 1 | 8.5 | NC |
| *Cheilosia urbana* | Syrphidae | 1 | 7 | NC |
| *Cheilosia vernalis* | Syrphidae | 1 | 6 | NC |
| *Cheilosia vulpina* | Syrphidae | 1 | 9 | NC |
| *Chelostoma campanularum* | Megachilidae | 2.9 | 6.5 | C |
| *Chelostoma distinctum* | Megachilidae | 1.8 | 7 | C |
| *Chelostoma florisomne* | Megachilidae | 2.3 | 9.5 | C |
| *Chelostoma rapunculi* | Megachilidae | 2.7 | 9 | C |
| *Chrysosomoxys macrocercus* | Tachinidae | 2.2 | 7.5 | NC |
| *Chrysotoxum bicinctum* | Syrphidae | 1 | 10.5 | NC |
| *Chrysotoxum intermedium* | Syrphidae | 1 | 12 | NC |
| *Chrysotoxum vernale* | Syrphidae | 1 | 11.5 | NC |
| *Chrysotoxum verralli* | Syrphidae | 1 | 11.5 | NC |
| *Coelioxys conoidea* | Megachilidae | 4.1 | 10 | NC |
| *Conops flavipes* | Conopidae | 4.2 | 11 | NC |
| *Conops quadrifasciatus* | Conopidae | 3.3 | 8.5 | NC |
| *Corynis crassicornis* | Cimbicidae | 0.9 | 8 | NC |
| *Crossocerus cinxius* | Crabronidae | 0.65 | 7 | C |
| *Crossocerus leucostoma* | Crabronidae | 0.6 | 8 | C |
| *Cylindromyia brassicaria* | Tachinidae | 2.4 | 9 | NC |
| *Dasysyrphus albostriatus* | Syrphidae | 1 | 9 | NC |
| *Didea alneti* | Syrphidae | 1 | 14 | NC |
| *Didea erratica* | Syrphidae | 1 | 12.5 | NC |
| *Dinera carinifrons* | Tachinidae | 2.3 | 7.5 | NC |
| *Dinera ferina* | Tachinidae | 3.5 | 12 | NC |
| *Dufourea alpina* | Halictidae | 1.3 | 5.5 | C |
| *Ectemnius borealis* | Crabronidae | 0.95 | 7 | C |
| *Ectemnius continuus* | Crabronidae | 1.4 | 10 | C |
| *Ectophasia crassipennis* | Tachinidae | 3.3 | 9.75 | NC |
| *Entomognathus brevis* | Crabronidae | 0.4 | 4.5 | C |
| *Epistrophe grossulariae* | Syrphidae | 1 | 13 | NC |
| *Epistrophe melanostoma* | Syrphidae | 1 | 11 | NC |
| *Epistrophe nitidicollis* | Syrphidae | 1 | 11.5 | NC |
| *Episyrphus balteatus* | Syrphidae | 1 | 11.5 | NC |
| *Eriozona syrphoides* | Syrphidae | 1.5 | 14 | NC |
| *Eristalinus taeniops* | Syrphidae | 1.5 | 12.5 | NC |
| *Eristalis arbustorum* | Syrphidae | 1 | 10 | NC |
| *Eristalis horticola* | Syrphidae | 1.5 | 12 | NC |
| *Eristalis interrupta* | Syrphidae | 1 | 12 | NC |
| *Eristalis jugorum* | Syrphidae | 1.5 | 12.5 | NC |
| *Eristalis pertinax* | Syrphidae | 1.5 | 14 | NC |
| *Eristalis rupium* | Syrphidae | 1 | 11.5 | NC |
| *Eristalis similis* | Syrphidae | 1.5 | 14 | NC |
| *Eristalis tenax* | Syrphidae | 1.5 | 15 | NC |
| *Erycia fatua* | Tachinidae | 2.1 | 8.5 | NC |
| *Eucera longicornis* | Apidae | 6.25 | 14.5 | C |
| *Eucera nigrescens* | Apidae | 8.25 | 13 | C |
| *Eulabidogaster setifacies* | Tachinidae | 2.1 | 7 | NC |
| *Eupeodes corollae* | Syrphidae | 1 | 8 | NC |
| *Eupeodes lapponicus* | Syrphidae | 1 | 8 | NC |
| *Eupeodes latifasciatus* | Syrphidae | 1 | 10 | NC |
| *Eupeodes luniger* | Syrphidae | 1 | 9.5 | NC |
| *Eupeodes tirolensis* | Syrphidae | 1 | 10 | NC |
| *Exorista rustica* | Tachinidae | 2.2 | 12 | NC |
| *Exorista tubulosa* | Tachinidae | 2.4 | 8.5 | NC |
| *Gorytes quinquecinctus* | Crabronidae | 0.7 | 10 | C |
| *Gymnosoma clavatum* | Tachinidae | 1.9 | 7 | NC |
| *Gymnosoma nitens* | Tachinidae | 1.7 | 5 | NC |
| *Gymnosoma rotundatum* | Tachinidae | 2.4 | 7.75 | NC |
| *Gymnosoma* sp. | Tachinidae | 2 | 7 | NC |
| *Halictus compressus* | Halictidae | 2.55 | 9.5 | C |
| *Halictus langobardicus* | Halictidae | 2.1 | 8.5 | C |
| *Halictus maculatus* | Halictidae | 1.6 | 8.5 | C |
| *Halictus rubicundus* | Halictidae | 2.23 | 10.5 | C |
| *Halictus scabiosae* | Halictidae | 3.65 | 13 | C |
| *Halictus simplex* | Halictidae | 2.75 | 9.75 | C |
| *Halictus subauratus* | Halictidae | 1.85 | 7 | C |
| *Helophilus pendulus* | Syrphidae | 1 | 12 | NC |
| *Helophilus trivittatus* | Syrphidae | 1 | 15.5 | NC |
| *Heriades rubicola* | Megachilidae | 1.8 | 6 | C |
| *Heriades truncorum* | Megachilidae | 2.2 | 8 | C |
| *Hoplitis adunca* | Megachilidae | 4.5 | 9 | C |
| *Hoplitis* sp. 1 | Megachilidae | 3.9 | 7.5 | C |
| *Hoplitis villosa* | Megachilidae | 4.8 | 12 | C |
| *Hyalurgus cruciger* | Tachinidae | 1.1 | 5.5 | NC |
| *Hylaeus brevicornis* | Colletidae | 0.75 | 4.75 | C |
| *Hylaeus communis* | Colletidae | 0.75 | 4.75 | C |
| *Hylaeus confusus* | Colletidae | 1 | 5.5 | C |
| *Hylaeus gibbus* | Colletidae | 1.1 | 6.75 | C |
| *Hylaeus hyalinatus* | Colletidae | 0.8 | 5.3 | C |
| *Hylaeus imparilis* | Colletidae | 0.55 | 4 | C |
| *Hylaeus punctatus* | Colletidae | 0.7 | 5 | C |
| *Hylaeus taeniolatus* | Colletidae | 0.6 | 4 | C |
| *Hylaeus tyrolensis* | Colletidae | 0.6 | 5 | C |
| *Hylaeus variegatus* | Colletidae | 1 | 6.25 | C |
| *Lasioglossum albipes* | Halictidae | 1.5 | 8 | C |
| *Lasioglossum angusticeps* | Halictidae | 1.8 | 7 | C |
| *Lasioglossum calceatum* | Halictidae | 2.2 | 10 | C |
| *Lasioglossum discum* | Halictidae | 2.6 | 8.75 | C |
| *Lasioglossum fulvicorne* | Halictidae | 1.3 | 7.5 | C |
| *Lasioglossum glabriusculum* | Halictidae | 1 | 4.5 | C |
| *Lasioglossum lativentre* | Halictidae | 1.7 | 8 | C |
| *Lasioglossum leucozonium* | Halictidae | 2.25 | 8 | C |
| *Lasioglossum malachurum* | Halictidae | 1.8 | 9 | C |
| *Lasioglossum morio* | Halictidae | 1.1 | 7 | C |
| *Lasioglossum nigripes* | Halictidae | 2.45 | 9.75 | C |
| *Lasioglossum parvulum* | Halictidae | 1.3 | 6.5 | C |
| *Lasioglossum politum* | Halictidae | 0.93 | 5.25 | C |
| *Lasioglossum punctatissimum* | Halictidae | 1.3 | 6 | C |
| *Lasioglossum pygmaeum* | Halictidae | 1 | 6 | C |
| *Lasioglossum rufitarse* | Halictidae | 1.3 | 6 | C |
| *Lasioglossum* sp. 1 | Halictidae | 1.2 | 6.5 | C |
| *Lasioglossum* sp. 2 | Halictidae | 2.5 | 8 | C |
| *Lasioglossum villosulum* | Halictidae | 1.65 | 6 | C |
| *Lasioglossum zonulum* | Halictidae | 2.5 | 8.5 | C |
| *Leucostoma simplex* | Tachinidae | 2.2 | 7 | NC |
| *Leucozona lucorum* | Syrphidae | 1 | 11.5 | NC |
| *Lindenius albilabris* | Crabronidae | 0.7 | 6 | C |
| *Linnaemya impudica* | Tachinidae | 4.3 | 13 | NC |
| *Linnaemya lithosiophaga* | Tachinidae | 1.7 | 7 | NC |
| *Linnaemya picta* | Tachinidae | 3.3 | 12 | NC |
| *Linnaemya zachvatkini* | Tachinidae | 3 | 12 | NC |
| *Macrophya montana* | Tenthredinidae | 0.95 | 11.25 | NC |
| *Macropis europaea* | Melittidae | 1.2 | 9 | C |
| *Masistylum arcuatum* | Tachinidae | 2.8 | 8.25 | NC |
| *Megachile circumcincta* | Megachilidae | 3.2 | 12 | C |
| *Megachile lagopoda* | Megachilidae | 6.5 | 18 | C |
| *Megachile leachella* | Megachilidae | 3.7 | 9 | C |
| *Megachile ligniseca* | Megachilidae | 4.5 | 14 | C |
| *Megachile melanopyga* | Megachilidae | 3.6 | 11 | C |
| *Megachile nigriventris* | Megachilidae | 6.2 | 15 | C |
| *Megachile pilidens* | Megachilidae | 3.7 | 8.75 | C |
| *Megachile sculpturalis* | Megachilidae | 5.65 | 20 | C |
| *Megachile* sp. 1 | Megachilidae | 4.8 | 15 | C |
| *Megachile* sp. 2 | Megachilidae | 4.1 | 12.5 | C |
| *Megachile versicolor* | Megachilidae | 4 | 9 | C |
| *Megachile willughbiella* | Megachilidae | 4.45 | 12.25 | C |
| *Megalodontes* sp. 1 | Megalodontesidae | 2.3 | 12 | NC |
| *Megalodontes* sp. 2 | Megalodontesidae | 2.2 | 12 | NC |
| *Melangyna compositarum* | Syrphidae | 1 | 10 | NC |
| *Melanogaster nuda* | Syrphidae | 0.6 | 5.5 | NC |
| *Melanostoma mellinum* | Syrphidae | 0.6 | 6 | NC |
| *Melanostoma scalare* | Syrphidae | 0.6 | 8.5 | NC |
| *Meliscaeva auricollis* | Syrphidae | 1 | 9.5 | NC |
| *Meliscaeva cinctella* | Syrphidae | 1 | 10 | NC |
| *Merodon aeneus* | Syrphidae | 1 | 8.5 | NC |
| *Merodon cinereus* | Syrphidae | 1 | 9 | NC |
| *Merodon costans* | Syrphidae | 1 | 11 | NC |
| *Merodon equestris* | Syrphidae | 1 | 13 | NC |
| *Merodon funestus* | Syrphidae | 1 | 9 | NC |
| *Merodon* sp. | Syrphidae | 1 | 10 | NC |
| *Mintho rufiventris* | Tachinidae | 1.4 | 7 | NC |
| *Myathropa florea* | Syrphidae | 1 | 12 | NC |
| *Neoascia podagrica* | Syrphidae | 0.6 | 5.5 | NC |
| *Nomada armata* | Apidae | 3.5 | 9 | NC |
| *Nomada flavopicta* | Apidae | 3.1 | 8 | NC |
| *Nomada sexfasciata* | Apidae | 4.7 | 11 | NC |
| *Nowickia ferox* | Tachinidae | 4.45 | 14.5 | NC |
| *Nowickia marklini* | Tachinidae | 4.3 | 13 | NC |
| *Osmia aurulenta* | Megachilidae | 4 | 12 | C |
| *Osmia bicolor* | Megachilidae | 5.2 | 11.5 | C |
| *Osmia caerulescens* | Megachilidae | 4.9 | 7.5 | C |
| *Osmia leaiana* | Megachilidae | 4 | 9 | C |
| *Osmia rufohirta* | Megachilidae | 4 | 7 | C |
| *Oxybelus mucronatus* | Crabronidae | 0.8 | 5 | C |
| *Oxybelus trispinosus* | Crabronidae | 1 | 5.5 | C |
| *Panurginus montanus* | Andrenidae | 1.4 | 7.5 | C |
| *Panzeria vivida* | Tachinidae | 2.7 | 8.5 | NC |
| *Paragus bicolor* | Syrphidae | 0.6 | 6.5 | NC |
| *Paragus constrictus* | Syrphidae | 0.6 | 5 | NC |
| *Paragus haemorrhous* | Syrphidae | 0.6 | 6.5 | NC |
| *Paragus* sp. | Syrphidae | 0.6 | 6 | NC |
| *Paragus tibialis* | Syrphidae | 0.6 | 6.5 | NC |
| *Parasyrphus lineolus* | Syrphidae | 1 | 9 | NC |
| *Peleteria iavana* | Tachinidae | 2.6 | 9 | NC |
| *Phasia aurulans* | Tachinidae | 2.1 | 6 | NC |
| *Phasia obesa* | Tachinidae | 1.6 | 5 | NC |
| *Physocephala rufipes* | Conopidae | 5 | 13 | NC |
| *Physocephala vittata* | Conopidae | 4.7 | 10.5 | NC |
| *Pipiza austriaca* | Syrphidae | 0.6 | 8.5 | NC |
| *Pipiza lugubris* | Syrphidae | 0.6 | 8 | NC |
| *Pipiza* sp. | Syrphidae | 0.6 | 8 | NC |
| *Pipizella divicoi* | Syrphidae | 0.6 | 6.5 | NC |
| *Pipizella* sp. | Syrphidae | 0.6 | 6 | NC |
| *Pipizella viduata* | Syrphidae | 0.6 | 6.5 | NC |
| *Platycheirus albimanus* | Syrphidae | 0.6 | 8.5 | NC |
| *Platycheirus scutatus* | Syrphidae | 0.6 | 8.5 | NC |
| *Platycheirus* sp. | Syrphidae | 0.6 | 8.5 | NC |
| *Platycheirus tarsalis* | Syrphidae | 0.6 | 8.5 | NC |
| *Polistes associus* | Vespidae | 1.5 | 14 | C |
| *Polistes biglumis* | Vespidae | 1.65 | 13 | C |
| *Polistes dominula* | Vespidae | 1.6 | 14 | C |
| *Polistes gallicus* | Vespidae | 1.6 | 14 | C |
| *Polistes nimpha* | Vespidae | 1.4 | 13.5 | C |
| *Polistes semenowi* | Vespidae | 1.5 | 13 | NC |
| *Prosena siberita* | Tachinidae | 5.9 | 8 | NC |
| *Psenulus pallipes* | Crabronidae | 0.5 | 6.5 | C |
| *Pseudapis diversipes* | Halictidae | 2.3 | 8.25 | C |
| *Pseudoanthidium scapulare* | Megachilidae | 3.75 | 6.5 | C |
| *Rhingia campestris* | Syrphidae | 3.5 | 9.5 | NC |
| *Rhodanthidium septemdentatum* | Megachilidae | 4.5 | 12.5 | C |
| *Rhogogaster picta* | Tenthredinidae | 0.6 | 12 | NC |
| *Scaeva pyrastri* | Syrphidae | 1.5 | 12.5 | NC |
| *Scaeva selenitica* | Syrphidae | 1.5 | 13.5 | NC |
| *Scolia hirta* | Scoliidae | 4.1 | 19 | NC |
| *Sicus ferrugineus* | Conopidae | 5.8 | 11.25 | NC |
| *Siphona flavifrons* | Tachinidae | 3.7 | 5.5 | NC |
| *Siphona geniculata* | Tachinidae | 3.05 | 5 | NC |
| *Solieria vacua* | Tachinidae | 1.75 | 6.75 | NC |
| *Sphaerophoria infuscata* | Syrphidae | 0.6 | 8.5 | NC |
| *Sphaerophoria interrupta* | Syrphidae | 0.6 | 8.5 | NC |
| *Sphaerophoria scripta* | Syrphidae | 0.6 | 11.5 | NC |
| *Sphaerophoria* sp. | Syrphidae | 0.6 | 9.5 | NC |
| *Sphaerophoria taeniata* | Syrphidae | 0.6 | 9 | NC |
| *Sphecodes gibbus* | Halictidae | 1.7 | 8 | NC |
| *Sphecodes monilicornis* | Halictidae | 2.1 | 9 | NC |
| *Sphecodes pellucidus* | Halictidae | 1.5 | 8 | NC |
| *Sphecodes schencki* | Halictidae | 1.7 | 8 | NC |
| *Sphegina clunipes* | Syrphidae | 0.6 | 6.5 | NC |
| *Sphegina* sp. | Syrphidae | 0.6 | 6.5 | NC |
| *Stelis punctulatissima* | Megachilidae | 3.1 | 8.5 | NC |
| *Strongygaster globula* | Tachinidae | 1 | 6 | NC |
| *Syritta pipiens* | Syrphidae | 0.6 | 8 | NC |
| *Syrphus ribesii* | Syrphidae | 1 | 11 | NC |
| *Syrphus torvus* | Syrphidae | 1 | 11.5 | NC |
| *Syrphus vitripennis* | Syrphidae | 1 | 9.5 | NC |
| *Tachina fera* | Tachinidae | 4.8 | 14 | NC |
| *Tachina magnicornis* | Tachinidae | 3.45 | 12.5 | NC |
| *Tenthredo arcuata* | Tenthredinidae | 1 | 10.5 | NC |
| *Tenthredo crassa* | Tenthredinidae | 1.05 | 13 | NC |
| *Tenthredo koehleri* | Tenthredinidae | 1.7 | 11.5 | NC |
| *Tenthredo olivacea* | Tenthredinidae | 0.9 | 11 | NC |
| *Tenthredo rubricoxis* | Tenthredinidae | 0.8 | 12 | NC |
| *Tenthredo* sp. 1 | Tenthredinidae | 0.9 | 10.5 | NC |
| *Tenthredo* sp. 2 | Tenthredinidae | 1 | 11 | NC |
| *Tenthredo zonula* | Tenthredinidae | 0.68 | 8.5 | NC |
| *Tenthredopsis* sp. 1 | Tenthredinidae | 0.9 | 13 | NC |
| *Tenthredopsis tischbeinii* | Tenthredinidae | 0.9 | 11 | NC |
| *Tetraloniella dentata* | Apidae | 4.3 | 12 | C |
| *Tetraloniella salicariae* | Apidae | 3 | 8 | C |
| *Thecophora atra* | Conopidae | 3 | 4.8 | NC |
| *Thecophora distincta* | Conopidae | 3.4 | 5.2 | NC |
| *Trachusa byssina* | Megachilidae | 4.45 | 10.5 | C |
| *Trichopoda pennipes* | Tachinidae | 2.4 | 8.5 | NC |
| *Volucella bombylans* | Syrphidae | 1.5 | 13 | NC |
| *Volucella pellucens* | Syrphidae | 1.5 | 15.5 | NC |
| *Xylocopa iris* | Apidae | 6 | 19 | C |
| *Xylocopa violacea* | Apidae | 7.85 | 22.5 | C |
| *Xylota jakutorum* | Syrphidae | 1 | 11 | NC |
| *Zodion cinereum* | Conopidae | 3.4 | 6 | NC |
| *Zophomyia temula* | Tachinidae | 2.7 | 11 | NC |

**Table S4** Traits of pollinator families: number of collected specimens (N), proboscis length in mm (mean and SD values of the measured specimens), body size in mm (mean and SD values of the measured specimens), and percentage of central and non-central forager species (*C* central-place foragers, *NC* non-central-place foragers)

| **Pollinator family** | **N** | **Proboscis (mm)** | **Body (mm)** | **C species (%)** | **NC species (%)** |
| --- | --- | --- | --- | --- | --- |
| Andrenidae | 86 | 1.73 (0.67) | 9.33 (2.17) | 100 | - |
| Apidae | 8045 | 6.88 (2.66) | 14.1 (3.56) | 78 | 22 |
| Cimbicidae | 2 | 0.85 (0.07) | 10 (2.83) | - | 100 |
| Colletidae | 37 | 0.79 (0.19) | 5.13 (0.88) | 100 | - |
| Conopidae | 43 | 4.1 (0.99) | 8.78 (3.12) | - | 100 |
| Crabronidae | 40 | 0.92 (0.4) | 7.62 (2.38) | 100 | - |
| Halictidae | 258 | 1.84 (0.62) | 7.87 (1.73) | 88 | 12 |
| Megachilidae | 116 | 3.9 (1.11) | 10.31 (3.32) | 94 | 6 |
| Megalodontesidae | 3 | 2.25 (0.07) | 12 (0) | - | 100 |
| Melittidae | 8 | 1.2 (NA) | 9 (NA) | 100 | - |
| Scoliidae | 4 | 4.1 (NA) | 19 (NA) | - | 100 |
| Syrphidae | 2000 | 0.98 (0.38) | 9.66 (2.49) | - | 100 |
| Tachinidae | 102 | 2.68 (1.07) | 8.74 (2.72) | - | 100 |
| Tenthredinidae | 81 | 0.91 (0.29) | 10.9 (1.76) | - | 100 |
| Vespidae | 16 | 1.54 (0.09) | 13.58 (0.49) | 83 | 17 |

**Table S5** Summary of the six global models. Abbreviated explanatory variables are honeybee abundance (*Apis*, ln-transformed), temperature (Temp), standardized functional richness of plant community (FRic), functional dispersion of plant community (FDis), trait similarity between wild pollinator community and the honeybee (TSim), proboscis length category (Prob), body size category (Body), type of foraging range (For), and taxonomic family (Fam). The continuous explanatory variables were scaled to mean 0 and standard deviation 1 to make slopes comparable

|  | Type of model | Response variable | Explanatory variables | Random effect |
| --- | --- | --- | --- | --- |
| Model 1 | Linear model | CWM resource overlap between wild pollinators and the honeybee (one value per network) | *Apis* + Temp + FRic + TSim + *Apis* × FRic + *Apis* × TSim + FRic × TSim + *Apis* × FRic × TSim | NA |
| Model 2 | Linear model | CWM resource overlap between wild pollinators and the honeybee (one value per network) | *Apis* + Temp + FDis + TSim + *Apis* × FDis + *Apis* × TSim + FDis × TSim + *Apis* × FDis × TSim | NA |
| Model 3 | Linear mixed-effect model | CWM resource overlap between wild pollinators and the honeybee (one value per network per trait category, i.e., proboscis shorter, similar, and longer than the honeybee) | *Apis* × Prob + Temp | Network |
| Model 4 | Linear mixed-effect model | CWM resource overlap between wild pollinators and the honeybee (one value per network per trait category, i.e., smaller, similar, and larger than the honeybee) | *Apis* × Body + Temp | Network |
| Model 5 | Linear mixed-effect model | CWM resource overlap between wild pollinators and the honeybee (one value per network per trait category, i.e., central forager and non-central forager) | *Apis* × For + Temp | Network |
| Model 6 | Linear mixed-effect model | CWM resource overlap between wild pollinators and the honeybee (one value per network per trait category, i.e., Andrenidae, Apidae, Colletidae, Conopidae, Crabronidae, Halictidae, Megachilidae, other families, Syrphidae, Tachinidae, Tenthredinidae, and Vespidae) | *Apis* × Fam + Temp | Network |

**Table S6** Results of the multi-model inference analysis testing the effects on CWM resource overlap of honeybee abundance (*Apis*, ln-transformed), temperature (Temp), standardized functional richness of plant community (FRic), trait similarity between wild pollinator community and the honeybee (TSim), and the following interactions: *Apis* × FRic, *Apis* × TSim, FRic × TSim, and *Apis* × FRic × TSim. The table reports the estimates for each variable, the ΔAICc, the R^2^, and the Akaike weight (w) for each model with ΔAICc < 6. All the explanatory variables were scaled to mean 0 and standard deviation 1

| **Ranking** | **Intercept** | ***Apis*** | **Temp** | **FRic** | **TSim** | ***Apis* × FRic** | ***Apis* × TSim** | **FRic × TSim** | ***Apis* × FRic × TSim** | **ΔAICc** | **R^2^** | **w** |
| --- | --- | --- | --- | --- | --- | --- | --- | --- | --- | --- | --- | --- |
| 1 | -1.471 | -0.087 | - | -0.010 | 0.143 | -0.193 | - | - | - | 0.000 | 0.290 | 0.286 |
| 2 | -1.474 | -0.084 | - | -0.008 | 0.155 | -0.201 | -0.058 | - | - | 1.917 | 0.300 | 0.110 |
| 3 | -1.458 | -0.087 | - | 0.000 | 0.159 | -0.190 | - | 0.043 | - | 2.290 | 0.295 | 0.091 |
| 4 | -1.472 | -0.089 | 0.007 | -0.007 | 0.144 | -0.191 | - | - | - | 2.682 | 0.290 | 0.075 |
| 5 | -1.472 | -0.087 | - | -0.056 | - | -0.189 | - | - | - | 3.373 | 0.202 | 0.053 |
| 6 | -1.517 | - | - | - | 0.158 | - | - | - | - | 3.511 | 0.120 | 0.049 |
| 7 | -1.517 | -0.084 | - | - | 0.152 | - | - | - | - | 3.884 | 0.154 | 0.041 |
| 8 | -1.458 | -0.083 | - | 0.006 | 0.177 | -0.199 | -0.069 | 0.055 | - | 4.065 | 0.310 | 0.037 |
| 9 | -1.474 | -0.084 | 0.000 | -0.007 | 0.155 | -0.201 | -0.058 | - | - | 4.741 | 0.300 | 0.027 |
| 10 | -1.517 | - | - | -0.061 | 0.139 | - | - | - | - | 4.931 | 0.136 | 0.024 |
| 11 | -1.459 | -0.089 | 0.007 | 0.003 | 0.160 | -0.188 | - | 0.043 | - | 5.099 | 0.295 | 0.022 |
| 12 | -1.517 | - | 0.048 | - | 0.159 | - | - | - | - | 5.226 | 0.131 | 0.021 |
| 13 | -1.517 | -0.093 | 0.061 | - | 0.152 | - | - | - | - | 5.270 | 0.172 | 0.021 |
| 14 | -1.517 | -0.075 | - | -0.043 | 0.139 | - | - | - | - | 5.890 | 0.161 | 0.015 |
| 15 | -1.471 | -0.084 | -0.015 | -0.062 | - | -0.194 | - | - | - | 5.894 | 0.203 | 0.015 |

**Table S7** Results of the multi-model inference analysis testing the effects on CWM resource overlap between wild pollinators and the honeybee of honeybee abundance (*Apis*, ln-transformed), temperature (Temp), functional dispersion of plant community (FDis), trait similarity between wild pollinator community and the honeybee (TSim), and the following interactions: *Apis* × FDis, *Apis* × TSim, FDis × TSim, and *Apis* × FDis × TSim. The table reports the estimates for each variable, the ΔAICc, the R^2^, and the Akaike weight (w) for each model with ΔAICc < 6. All the explanatory variables were scaled to mean 0 and standard deviation 1

| Ranking | Intercept | *Apis* | Temp | FDis | TSim | *Apis* × FDis | *Apis* × TSim | FDis × TSim | *Apis* × FDis × TSim | ΔAICc | R^2^ | w |
| --- | --- | --- | --- | --- | --- | --- | --- | --- | --- | --- | --- | --- |
| 1 | -1.517 | - | - | - | 0.171 | - | - | - | - | 0.000 | 0.141 | 0.144 |
| 2 | -1.517 | -0.082 | - | - | 0.165 | - | - | - | - | 0.436 | 0.173 | 0.116 |
| 3 | -1.517 | - | - | -0.082 | 0.143 | - | - | - | - | 0.635 | 0.169 | 0.105 |
| 4 | -1.517 | -0.087 | - | -0.088 | 0.134 | - | - | - | - | 0.842 | 0.205 | 0.095 |
| 5 | -1.517 | - | 0.038 | - | 0.170 | - | - | - | - | 1.944 | 0.148 | 0.055 |
| 6 | -1.517 | -0.090 | 0.052 | - | 0.161 | - | - | - | - | 2.128 | 0.185 | 0.050 |
| 7 | -1.519 | -0.081 | - | - | 0.168 | - | -0.023 | - | - | 2.793 | 0.174 | 0.036 |
| 8 | -1.527 | - | - | -0.067 | 0.140 | - | - | -0.030 | - | 2.857 | 0.173 | 0.035 |
| 9 | -1.519 | -0.079 | - | -0.084 | 0.135 | -0.059 | - | - | - | 2.967 | 0.212 | 0.033 |
| 10 | -1.517 | -0.100 | - | -0.134 | - | - | - | - | - | 2.981 | 0.130 | 0.033 |
| 11 | -1.517 | - | 0.021 | -0.077 | 0.144 | - | - | - | - | 2.982 | 0.171 | 0.032 |
| 12 | -1.517 | -0.092 | 0.034 | -0.080 | 0.135 | - | - | - | - | 3.097 | 0.210 | 0.031 |
| 13 | -1.527 | -0.088 | - | -0.072 | 0.130 | - | - | -0.031 | - | 3.135 | 0.210 | 0.030 |
| 14 | -1.518 | -0.086 | - | -0.087 | 0.137 | - | -0.019 | - | - | 3.344 | 0.206 | 0.027 |
| 15 | -1.517 | - | - | -0.131 | - | - | - | - | - | 3.344 | 0.083 | 0.027 |
| 16 | -1.518 | -0.089 | 0.050 | - | 0.164 | - | -0.016 | - | - | 4.650 | 0.186 | 0.014 |
| 17 | -1.518 | -0.092 | - | -0.131 | - | -0.056 | - | - | - | 5.077 | 0.136 | 0.011 |
| 18 | -1.517 | -0.104 | 0.030 | -0.127 | - | - | - | - | - | 5.205 | 0.134 | 0.011 |
| 19 | -1.528 | - | 0.026 | -0.058 | 0.140 | - | - | -0.034 | - | 5.245 | 0.176 | 0.010 |
| 20 | -1.530 | -0.093 | 0.040 | -0.059 | 0.130 | - | - | -0.039 | - | 5.377 | 0.217 | 0.010 |
| 21 | -1.529 | -0.079 | - | -0.069 | 0.131 | -0.058 | - | -0.031 | - | 5.390 | 0.216 | 0.010 |
| 22 | -1.517 | -0.096 | - | - | - | - | - | - | - | 5.456 | 0.044 | 0.009 |
| 23 | -1.522 | -0.076 | - | -0.082 | 0.140 | -0.069 | -0.032 | - | - | 5.464 | 0.215 | 0.009 |
| 24 | -1.517 | - | - | - | - | - | - | - | - | 5.477 | 0.000 | 0.009 |
| 25 | -1.518 | -0.083 | 0.023 | -0.079 | 0.135 | -0.047 | - | - | - | 5.535 | 0.214 | 0.009 |
| 26 | -1.517 | - | 0.015 | -0.128 | - | - | - | - | - | 5.646 | 0.084 | 0.009 |
| 27 | -1.530 | -0.087 | - | -0.070 | 0.134 | - | -0.025 | -0.034 | - | 5.710 | 0.212 | 0.008 |
| 28 | -1.518 | -0.091 | 0.032 | -0.080 | 0.137 | - | -0.015 | - | - | 5.747 | 0.211 | 0.008 |

**Table S8** Results of the multi-model inference analysis for single traits of pollinators, testing the effects on CWM resource overlap between wild pollinators and the honeybee of honeybee abundance (*Apis*, ln-transformed), temperature (Temp), and **a** proboscis length (Prob) and the interaction between honeybee abundance and proboscis length (*Apis* × Prob), **b** body size (Body) and the interaction between honeybee abundance and body size (*Apis* × Body), **c** type of foraging range (For) and the interaction between honeybee abundance and type of foraging range (*Apis* × For), and **d** taxonomic family (Fam) and the interaction between honeybee abundance and taxonomic family (*Apis* × Fam). The table reports the estimate for each continuous variable or the presence of each categorical variable in the model, the ΔAICc, the R^2^, and the Akaike weight (w) for each model of the set with ΔAICc < 6. All the continuous explanatory variables were scaled to mean 0 and standard deviation 1

| **(a) Proboscis length** | | |  |  |  |  |  |  |
| --- | --- | --- | --- | --- | --- | --- | --- | --- |
| **Ranking** | **Intercept** | ***Apis*** | **Temp** | **Prob** | ***Apis* × Prob** | **ΔAICc** | **R^2^** | **w** |
| 1 | -1.675 | - | - | + | - | 0.000 | 0.117 | 0.487 |
| 2 | -1.674 | -0.032 | - | + | - | 1.918 | 0.119 | 0.187 |
| 3 | -1.676 | - | 0.025 | + | - | 2.017 | 0.118 | 0.178 |
| 4 | -1.675 | -0.036 | 0.030 | + | - | 3.911 | 0.120 | 0.069 |
| 5 | -1.672 | -0.099 | - | + | + | 4.893 | 0.128 | 0.042 |
| 6 | -1.673 | -0.104 | 0.032 | + | + | 6.916 | 0.129 | 0.015 |
| **(b) Body size category** | | |  |  |  |  |  |  |
| **Ranking** | **Intercept** | ***Apis*** | **Temp** | **Body** | ***Apis* × Body** | **ΔAICc** | **R^2^** | **w** |
| 1 | -1.987 | - | 0.087 | + | - | 0.000 | 0.249 | 0.339 |
| 2 | -1.986 | - | - | + | - | 0.533 | 0.236 | 0.260 |
| 3 | -1.985 | -0.061 | 0.096 | + | - | 0.811 | 0.257 | 0.226 |
| 4 | -1.985 | -0.048 | - | + | - | 1.899 | 0.240 | 0.131 |
| 5 | -1.987 | -0.047 | 0.096 | + | + | 5.025 | 0.258 | 0.027 |
| 6 | -1.986 | -0.034 | - | + | + | 6.085 | 0.241 | 0.016 |
| **(c) Type of foraging range** | | |  |  |  |  |  |  |
| **Ranking** | **Intercept** | ***Apis*** | **Temp** | **For** | ***Apis* × For** | **ΔAICc** | **R^2^** | **w** |
| 1 | -1.394 | -0.092 | - | + | - | 0.000 | 0.135 | 0.343 |
| 2 | -1.394 | - | - | + | - | 0.653 | 0.110 | 0.248 |
| 3 | -1.395 | -0.098 | 0.041 | + | - | 1.674 | 0.140 | 0.149 |
| 4 | -1.394 | -0.085 | - | + | + | 2.236 | 0.135 | 0.112 |
| 5 | -1.395 | - | 0.027 | + | - | 2.624 | 0.112 | 0.092 |
| 6 | -1.395 | -0.091 | 0.041 | + | + | 3.959 | 0.140 | 0.047 |
| **(d) Taxonomic family** | | |  |  |  |  |  |  |
| **Ranking** | **Intercept** | ***Apis*** | **Temp** | **Fam** | ***Apis* × Fam** | **ΔAICc** | **R^2^** | **w** |
| 1 | -1.987 | -0.074 | 0.081 | + | - | 0.000 | 0.239 | 0.393 |
| 2 | -1.993 | - | 0.071 | + | - | 1.049 | 0.230 | 0.232 |
| 3 | -1.988 | -0.065 | - | + | - | 1.401 | 0.230 | 0.195 |
| 4 | -1.992 | - | - | + | - | 1.583 | 0.223 | 0.178 |

**Fig. S1** Map of the 51 sampling sites. Map credit: © OpenStreetMap contributors


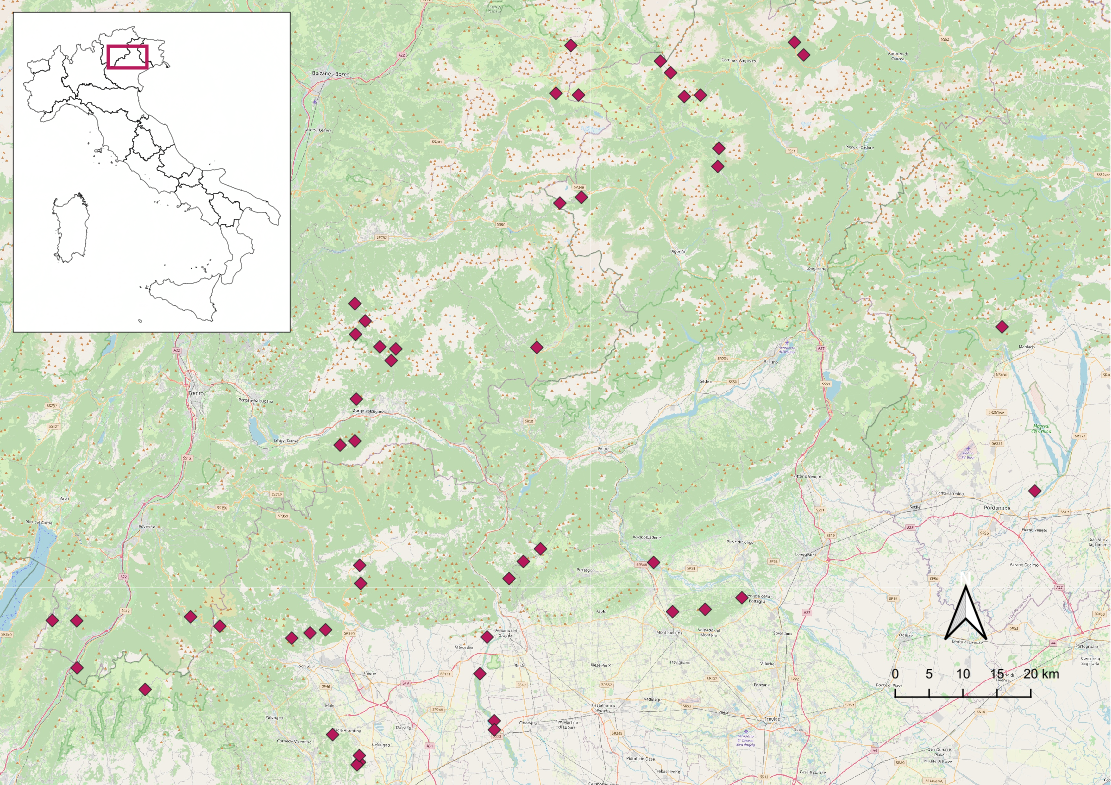


**Fig. S2** Plots showing the relationships between functional traits of pollinators, i.e., **a** proboscis length and body size, **b** type of foraging range and proboscis length, and **c** type of foraging range and body size. In all plots, proboscis length and body size were log-transformed

**
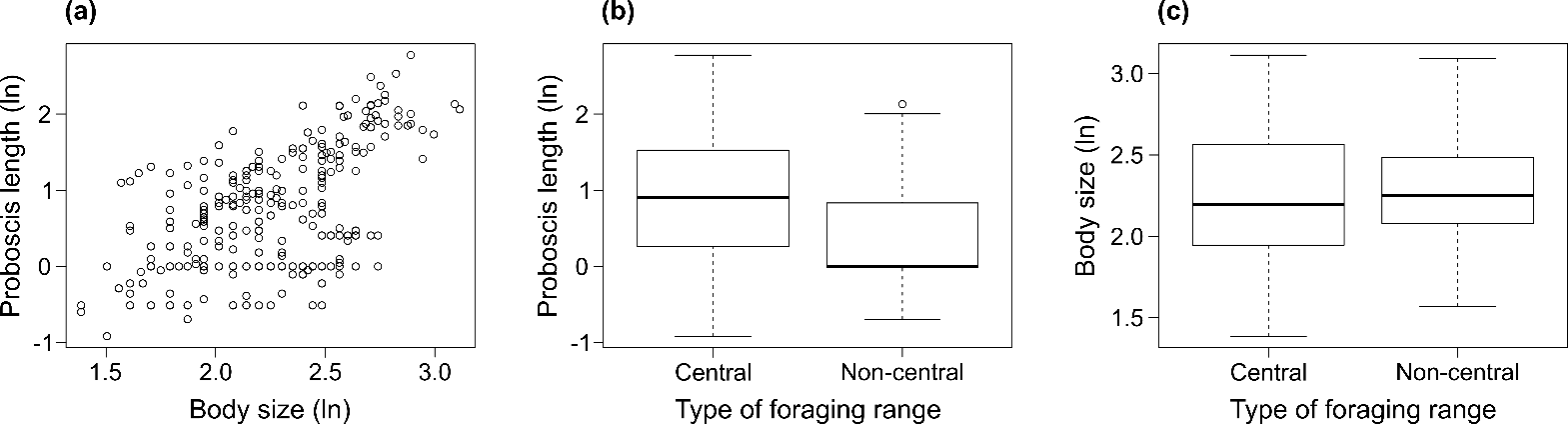
**

**Fig. S3** Model estimates from the model-averaging procedure based on the set of models with all functional traits of both plants and pollinators. Explanatory variables of the global model are honeybee abundance (*Apis*, ln-transformed), temperature (Temp), functional dispersion of plant community (FDis), trait similarity between wild pollinator community and the honeybee (TSim), and the following interactions: *Apis* × FDis, *Apis* × TSim, FDis × TSim, and *Apis* × FDis × TSim. All explanatory variables were scaled to mean 0 and standard deviation 1. Dots indicate the model estimated means, while error bars indicate the 95% confidence intervals for the expected value of the variable. The variable effect is supported when the confidence intervals do not include zero


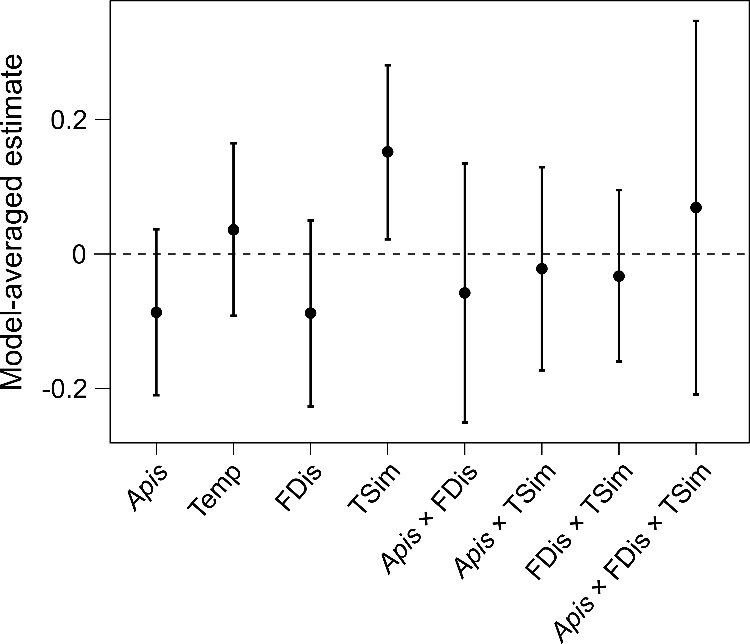


**Additional references for pollinator functional traits**

Alford DV (1975) Bumblebees. Davis-Poynter, London

Amiet F, Müller A, Neumeyer R (2014) Fauna Helvetica 4, Apidae 2 – *Colletes*, *Dufourea*, *Hylaeus*, *Nomia*, *Nomioides*, *Rhophitoides*, *Rophites*, *Sphecodes*, *Systropha*. Centre Suisse de Cartographie de la Faune & Schweizerische Entomologische Gesellschaft, Neuchâtel

Amiet F, Herrmann M, Müller A, et al (2001) Fauna Helvetica 6, Apidae 3 – *Halictus*, *Lasioglossum*. Centre Suisse de Cartographie de la Faune & Schweizerische Entomologische Gesellschaft, Neuchâtel

Amiet F, Herrmann M, Müller A, et al (2004) Fauna Helvetica 9, Apidae 4 – *Anthidium*, *Chelostoma*, *Coelioxys*, *Dioxys*, *Heriades*, *Lithurgus*, *Megachile*, *Osmia*, *Stelis*. Centre Suisse de Cartographie de la Faune & Schweizerische Entomologische Gesellschaft, Neuchâtel

Bitsch J, Barbier Y, Gayubo SF, et al (1997) Hyménoptères Sphecidae d'Europe occidentale, Volume 2. Faune de France, 82

Bitsch J, Dollfuss H, Bouček ZRJ, et al (2007) Hyménoptères Sphecidae d'Europe occidentale, Volume 3. Faune de France, 86

Bitsch J, Leclercq J (1993) Hyménoptères Sphecidae d'Europe occidentale, Volume 1. Faune de France, 79

Cariveau DP, Nayak GK, Bartomeus I, et al (2016) The allometry of bee proboscis length and its uses in ecology. PLoS ONE 11:1-13

Ebmer AW (1969) Die bienen des genus *Halictus* Latr. s.l. im Grosraum von Linz (Hymenoptera Apidae). Systematik, biogeographie, ökologie und biologie mit berücksichtingung aller bisher aus Mitteleuropa bekannten Arten. Teile I-III. Naturkundliches Jahrbuch der Stadt Linz 15:133-183

von Hagen E (1994) Hummeln: bestimmen, ansiedeln, vermehren, schützen. Naturbuch Vlg., Augsburg

Scheuchl E, Willner W (2016) Taschenlexicon der Wildbienen Mitteleuropas. Quelle & Meyer Verlag, Wiebelsheim

Schmid-Egger C, Scheuchl E (1997) Illustrierte bestimmungstabellen der wilbienen Deutschlands un Österreichs, band 3 – Andrenidae. Norbert Präbst Satz & Druck
